# Elucidating the crosstalk mechanism between IFN-gamma and IL-6 via mathematical modelling

**DOI:** 10.1186/1471-2105-14-41

**Published:** 2013-02-06

**Authors:** Yun-feng Qi, Yan-xin Huang, Hong-yan Wang, Yu Zhang, Yong-li Bao, Lu-guo Sun, Yin Wu, Chun-lei Yu, Zhen-bo Song, Li-hua Zheng, Ying Sun, Guan-nan Wang, Yu-xin Li

**Affiliations:** 1 National Engineering Laboratory for Druggable Gene and Protein Screening, Northeast Normal University, 130024, Changchun, China; 2 Research Center of Agriculture and Medicine Gene Engineering of Ministry of Education, Northeast Normal University, 130024, ChangChun, China

## Abstract

**Background:**

Interferon-gamma (IFN-gamma) and interleukin-6 (IL-6) are multifunctional cytokines that regulate immune responses, cell proliferation, and tumour development and progression, which frequently have functionally opposing roles. The cellular responses to both cytokines are activated via the Janus kinase/signal transducer and activator of transcription (JAK/STAT) pathway. During the past 10 years, the crosstalk mechanism between the IFN-gamma and IL-6 pathways has been studied widely and several biological hypotheses have been proposed, but the kinetics and detailed crosstalk mechanism remain unclear.

**Results:**

Using established mathematical models and new experimental observations of the crosstalk between the IFN-gamma and IL-6 pathways, we constructed a new crosstalk model that considers three possible crosstalk levels: (1) the competition between STAT1 and STAT3 for common receptor docking sites; (2) the mutual negative regulation between SOCS1 and SOCS3; and (3) the negative regulatory effects of the formation of STAT1/3 heterodimers. A number of simulations were tested to explore the consequences of cross-regulation between the two pathways. The simulation results agreed well with the experimental data, thereby demonstrating the effectiveness and correctness of the model.

**Conclusion:**

In this study, we developed a crosstalk model of the IFN-gamma and IL-6 pathways to theoretically investigate their cross-regulation mechanism. The simulation experiments showed the importance of the three crosstalk levels between the two pathways. In particular, the unbalanced competition between STAT1 and STAT3 for IFNR and gp130 led to preferential activation of IFN-gamma and IL-6, while at the same time the formation of STAT1/3 heterodimers enhanced preferential signal transduction by sequestering a fraction of the activated STATs. The model provided a good explanation of the experimental observations and provided insights that may inform further research to facilitate a better understanding of the cross-regulation mechanism between the two pathways.

## Background

The Janus kinase/signal transducer and activator of transcription (JAK/STAT) pathway can be activated by a wide range of cytokines. The binding of cytokines to their receptors drives receptor dimerization and phosphorylation, which leads to the recruitment, activation and dimerization of STATs [1-5]. STAT homodimers are translocated to the nucleus, where they regulate the expression of target genes [6,7]. IFN-gamma was first identified in a mitogen–activated lymphocyte supernatant based on its distinctive antiviral activity. It is widely involved in protection against tumour development and cancer immunoediting [4]. IFN-gamma triggers the prolonged activation of the transcription factor STAT1 via the IFN-gamma receptor (IFNR) and JAK, which induces target gene expression by binding to the gamma-activated sequences (GAS) in the promoters of these genes [6]. Thus, the activation of the IFN-gamma/JAK/STAT1 pathway can prevent the expansion of many different normal and neoplastic cells [8]. However, IL-6 also has important roles in triggering the acute phase response of the body to injury or inflammation [2]. The receptor involved with the recognition of IL-6 can be subdivided into the non-signalling part, i.e., glycoprotein 80 (gp80), and the signalling part, i.e., glycoprotein 130 (gp130). IL-6 attaches to gp80 and drives IL6-gp80 complex binding to the gp130-JAK complex, which forms the IL6-gp80-gp130-JAK complex. gp130 associates with JAK and it is tyrosine-phosphorylated in response to IL-6 stimulation, which leads to the activation of the JAK/STAT3 pathway and MAPK (mitogen-activated protein kinase) cascades [2,9]. IL-6 activates target genes involved with differentiation, survival, apoptosis and proliferation, and it plays important roles in pro- and anti-inflammatory functions, acute phase and immune responses in the organism, and tumour progression [2,6]. Three types of negative regulators are involved in the regulation of the IFN-gamma and IL-6 pathways: the suppressor of cytokine signalling (SOCS), SH2 domain-containing tyrosine phosphates 2 (SHP-2) and various cytoplasmic and nuclear protein tyrosine phosphates (PPs) [10,11]. SOCS1 and SOCS3 are induced by JAK/STAT pathway and they bind to the activated receptors of IFN-gamma and IL-6, respectively, which negatively regulate the signal transduction of IFN-gamma and IL-6 [12,13]. SHP-2 acts as a phosphatase in activated receptor complexes of IFN-gamma and IL-6 and it negatively regulates the activation of STATs [14,15]. PP1 dephosphorylates STAT*s (* represents species-activation states in this study) in the cytoplasm, while PP2 dephosphorylates STAT*s in the nucleus, which results in STATs being returned to the cytosol, thereby influence the activation of STATs [16,17].

Previous studies have indicated that IFN-gamma and IL-6 have opposing roles in cell proliferation, apoptotic death and inflammation, which are closely related to the specific patterns and duration of STAT activation after their stimulation [6]. IFN-gamma mainly phosphorylates STAT1, which has many pro-inflammatory effects [8,18,19]. By contrast, IL-6 is a potent activator of STAT3, which contributes to its anti-inflammatory functions [2,20,21]. However, the detailed molecular mechanism leading to the unbalanced activation of STATs after IFN-gamma and IL-6 stimulation remains unclear. Qing et al. suggested that tyrosine 419 in the IFN-gamma receptor subunit 1(IFNGR1) is required for the activation of both STAT1 and STAT3 [19]. In response to IL-6, STAT3 binds to phosphorylated (p)YXXQ motifs (Y767,Y814,Y905 and Y915) of gp130, whereas STAT1 is recruited to a more restricted consensus sequence pYXPQ (Y905 and Y915) in gp130 [2]. In addition, researchers have provided some interesting experimental results using STAT-deficient cells. Qing et al. showed that the activation of STAT3 in response to IFN-gamma was much stronger and more prolonged in STAT1-null cells than wild-type cells [19]. Costa-Pereira et al. showed that IL-6 mediated an IFN-gamma-like response in mouse embryo fibroblasts lacking STAT3, including the prolonged activation of STAT1 and it promoted the induction of multiple IFN-gamma-inducible genes [20]. However, Regis et al. reported that the activation of STAT1 in human neoplastic T lymphocytes after IFN-gamma stimulation was generally unaffected by STAT3 silencing [22]. Ho et al. argued that STAT3 did not affect the activation of STAT1 [23]. Bluyssen et al. reported that pre-treatment of EC with IFN-gamma significantly reduced the activation of STAT3 after induction by IL-6, but without affecting the total amounts of STAT3 [18]. However, Kaur et al. reported that the activation of STAT1 by IFN-gamma was mainly unaffected after pre-treatment with IL-6 or other gp130-related cytokines in SH-SY5Y human neuroblastoma cells [24]. These experimental results indicate that STAT1 and STAT3 may have common binding sites within IFN-gamma and IL-6 receptors, while the activation of STAT3 may depend on the concentration of STAT1 and vice versa. Moreover, the interactions between IFN-gamma and IL-6 signals are not symmetric.

Thyrell et al. also reported that IFN-alpha could influence the signal response of IL-6 in multiple myeloma, which resulted in a decrease in STAT3 homodimer DNA-binding activity and a shift from STAT3 homodimers to STAT1/3 heterodimers [25]. Herrero et al. observed that pre-treatment with IFN-gamma could affect the signal response of IL-10 in macrophages, which caused the IL-10 mediated STAT activation pattern to switch from STAT3 homodimers to STAT1/3 heterodimers [26]. Therefore, changes between STAT homodimers and STAT1/3 heterodimers may represent a biologically relevant approach to determining the crosstalk between IFN-gamma and IL-6 pathways. However, how the formation of STAT1/3 heterodimers regulates the interactions between IFN-gamma and IL-6 signals is not fully understood.

Systems biology modelling generally aims to find fairly plausible mechanistic models that include all of the relevant key processes in a biochemical system. It is considered to be a powerful analytical approach to understanding the essential mechanisms of the physiological functions of normal tissues and pathological progression during complex diseases [27]. The first systems biology model of the IFN-gamma/JAK/STAT1 pathway was developed by Yamada et al. [28]. They modelled activation of the JAK/STAT1 pathway in response to IFN-gamma and analysed the effects of the feedback loop regulated by SOCS1. Later, Zi et al. conducted a multi-parametric sensitivity analysis of the model produced by Yamada et al. and indicated that the concentrations of SOCS1, nuclear phosphatase PP2 and cytoplasmic STAT1, as well as some of the reaction steps that affected those concentrations, were the most sensitive to perturbation [29]. The first model of the IL-6/JAK/STAT3 pathway was produced by Singh et al. [30]. Recently, Moya et al. proposed an updated model of IL-6 and IL-10 signalling via JAK/STAT and ERK-C/EBPβ activation [31]. The model was used to investigate dynamical features of the system such as the activity ratio of JAK/STAT and ERK-C/EBPβ with different stimulation levels of IL-6 and IL-10. The dynamic behaviours of some individual molecules, such as STATs and SOCSs, in the IFN-gamma and IL-6 pathways were investigated in previous studies [28,30,31], but signalling of the crosstalk during signal transduction by IFN-gamma and IL-6 has still not been modelled.

In this study, we developed a crosstalk model of the IFN-gamma and IL-6 pathways by combining previously established mathematical models and by comprehensively analyzing the interactions between the two pathways. The model considered three possible levels of crosstalk between the two pathways: (1) the competition between STAT1 and STAT3 for IFNR and gp130; (2) the mutual negative regulation between IFN-gamma and IL-6 via the regulators SOCS1 and SOCS3; and (3) the restrictive effects of the formation of STAT1/3 heterodimers on the activation of the transcription factors STAT1 and STAT3. We considered a number of protocols where cells were stimulated by IFN-gamma and/or IL-6. The simulation results showed that the model provided a good explanation of the experimental observations and it provided new insights that could inform further research to facilitate a better understanding of the cross-regulation between the IFN-gamma and IL-6 pathways.

## Results

### Model description

Based on the model of the IFN-gamma/JAK/STAT1 pathway produced by Yamada et al. [28] and the model of the IL-6/10/JAK/STAT3 pathway produced by Moya et al. [31], we established a crosstalk model of the IFN-gamma and IL-6 pathways. A schematic diagram of the model is shown in Additional file 1: Figure S1. In this model, the components of the two previous mathematical models, their structures and most of the parameters were left unchanged. For simplicity, we specified that SHP-2 could repress the activated receptors of IFN-gamma and IL-6, while PP1 and PP2 (PPX and PPN in Yamada et al.’s work) could dephosphorylate STAT1 and STAT3 in the cytoplasm and the nucleus, respectively. We removed any reactions and components that were not connected with IFN-gamma and IL-6 signalling, such as IL-10. Sixteen new reactions were added based on the possible mechanisms of cross-regulation between IFN-gamma and IL-6.

The structure of the STAT1 and STAT3 proteins contains an oligomerization domain, a coiled-coil domain, a DNA-binding domain, a linker domain, an SH2 domain and a transactivation domain [32,33]. The recruitment of STAT1 and STAT3 to the activated receptor complexes is known to be mediated by their SH2 domains and phosphorylation of the receptor tyrosine motifs is required [34-36]. After receptor binding, the STATs are phosphorylated on a single tyrosine residue (Tyr 701 in STAT1 and Tyr705 in STAT3) [37,38]. Many experimental observations have shown that STAT1 and STAT3 may combine with the same docking sites in IFNR and gp130 [2,19]. Thus, we hypothesized that STAT1 and STAT3 might compete for the same phosphorylated docking sites in IFNR and gp130, via their SH2 domains in our model. After STAT1 and STAT3 combine with the activated receptors complexes via IFN-gamma and/or IL-6, they are phosphorylated and disassociate from the receptors. Based on these considerations, the new biochemical reactions (N1) - (N3) and (N4) - (N6) (see “New biochemical reactions added to the crosstalk model” in Additional file 1) were added to our model to simulate the activation of STAT3 after IFN-gamma stimulation and the activation of STAT1 after IL-6 stimulation, respectively.

The kinetic parameters of these new reactions are important because they reflect the properties of the biological system. Wiederkehr-Adam et al. indicated that the SH2 domain of STAT1 had a much higher affinity for the phosphotyrosine 419 motif in IFNGR1 than that in STAT3 [39]. After IL-6 stimulation, STAT3 binds to the flexible pYXXQ motifs in gp130, whereas STAT1 is recruited to the more restricted consensus sequence of pYXPQ in gp130 [2]. Based on these observations, we hypothesized the unbalanced competitive binding of STAT1 and STAT3 with IFNR and gp130 after IFN-gamma and IL-6 stimulation, respectively. Additional file 1: Tables S1-S3 show that the main effector of IL-6 signalling, STAT3, had a higher affinity for gp130 than STAT1. Similarly, the main effector of IFN-gamma signalling, STAT1, had a higher affinity for IFNR than STAT3.

SHP-2 and SOCS combine to regulate signal transduction by IFN-gamma and IL-6 [1,12,40]. SOCS1 inhibits the JAK/STAT pathway by binding to the activation loop of JAK via its SH2 domain [12]. SOCS3 can also bind to JAK [41]. SOCS1 and SHP-2 combine with different sites in the receptor complexes of IFN-gamma [28]. However, SOCS3 and SHP-2 may have similar binding specificities. Experiments have suggested that SOCS3 and SHP2 may compete for same site (tyrosine Y759) in gp130 after IL-6 stimulation [42,43]. In our model, SOCS1 and SHP-2 were capable of binding to the receptor complex of IFN-gamma without mutual interference, whereas SOCS3 and SHP-2 could competitively bind to the receptor complex. Distinct genes belonging to the SOCS family are induced as immediate early genes (IEGs) downstream of different STATs and they can inhibit STAT activation in a classical negative feedback loop [44]. It is generally recognized that SOCS1 has an important role in modulating IFN-gamma signalling [45], whereas SOCS3 mainly affects IL-6 signalling [46]. It is also well known that the STAT1 and STAT3 homodimers are direct transcription factors of the JAK/STAT pathway, which play important roles in signal transduction during IFN and gp130 receptor signalling [6]. However, Bluyssen et al. reported that SOCS3 could also be induced after IFN-gamma stimulation in EC and that it could inhibit signal transduction by IL-6 [18]. Qing et al. observed that the STAT1 and STAT3 homodimers could both be induced after IFN-gamma stimulation in MEFs, which both bound to the same GAS element in the SOCS3 promoter [19]. The sequence of this GAS element is conserved in mice, rats and humans [47]. It was shown that STAT3 activation was much stronger and more prolonged in STAT1-null cells, and that SOCS3 was strongly induced in wild-type and STAT1-null cells, while the levels of SOCS3 mRNA were greatly increased in STAT3-null cells [19]. Thus, it is speculated that STAT1 homodimers might also promote the transcription of SOCS3 in the same way as STAT3 homodimers. However, no experimental evidence indicates that STAT3 homodimers can combine with the promoter region of SOCS1. Thus, our model does not regard STAT3 homodimers as an efficient transcription factor for SOCS1. We added equation (N7) to our model to simulate the transcription of SOCS3 mRNA after its induction by STAT1 homodimers in the nucleus, which is represented as (STAT1N*)2.

Thyrell et al. reported that IFN-alpha could affect the signal response of IL-6 in multiple myeloma, which resulted in a decrease in STAT3 homodimer DNA-binding activity and a shift from STAT3 homodimers to STAT1/3 heterodimers [25]. Herrero et al. showed that pre-treatment with IFN-gamma could affect the signal response of IL-10 in macrophages, causing the IL-10-mediated activation pattern to switch from STAT3 homodimers to STAT1/3 heterodimers [26]. These experimental results showed that STAT1/3 heterodimers play important roles in the crosstalk between different cytokines. The activation of STATs after cytokine stimulation led to the formation of STAT homo- and heterodimers [2,6].

Haan et al. reported that IL-6 stimulation of primary human macrophages led to a different distribution of STAT dimer species in the cytosol and nucleus. In particular, they showed that STAT1/3 heterodimers were present in the cytosol and nucleus [48]. The size of STATs exceeds 90 kDa, which is far beyond the exclusion limit of the nuclear pores, so STATs need to be translocated actively into the nucleus [2,6]. Tyrosine phosphorylation is not necessarily required for STAT nuclear translocation [49]. Basic residues (Lys410 and Arg413) are known to contribute to the nuclear localization signals (NLSs) of dimeric STAT1 [50]. PP1 dephosphorylates STAT*s in the cytoplasm, while PP2 dephosphorylates STAT*s in the nucleus, which leads to STATs being returned to the cytosol [16,17,51]. It was postulated that the nuclear export signals (NESs) in the DNA-binding domain of STAT1 is comprised of the residues 399–410 [52]. The formation and dephosphorylation of STAT1 and STAT3 homodimers in the cytosol and nucleus were modelled by Yamada et al. and Moya et al., respectively. Their models also described the translocation of STAT1 and STAT3 homodimers from the cytosol to the nucleus, and the export of dephosphorylated STAT monomer from the nucleus to the cytoplasm [28,31]. In our model, biochemical reactions (N8) - (N9) were added to simulate the formation of STAT1/3 heterodimers in the cytoplasm and nuclei. For simplicity, it was supposed that only STAT1* and STAT3* could form STAT1/3 heterodimers. Biochemical reaction (N10) was added to simulate the STAT1/3 heterodimer translocation process from the cytoplasm to the nucleus based on the translocation of STAT homodimers. It was also assumed that STAT1/3 heterodimers could be dephosphorylated by PP1 and PP2, which resulted in STAT export from the nucleus to the cytoplasm. Biochemical reactions (N11)-(N16) were added to simulate this process. The specific biological role of STAT1/3 heterodimers remains obscure [6], so we did not regard the STAT1/3 heterodimers as efficient transcription factors in our model.

To construct the crosstalk model, we merged the common components from previous models, such as SHP-2, PP1 and PP2. JAK1 and JAK2 are two species in the JAK family, which play important roles in the signal responses of IFN-gamma and IL-6 [40]. In previous studies, JAK1 and JAK2 were treated as JAK for simplicity [28,31]. JAK1 and JAK2 can combine with the receptors of IFN-gamma and IL-6, but the signal transduction activities of IFN-gamma and IL-6 may relate to specific types of JAKs. The internal membrane proximal regions of JAK1 and JAK2,which responds to the IFN-gamma signal, bind the IFN-gamma receptor subunits IFNGR1 and IFNGR2 [53]. During IL-6 signalling, JAK1 and JAK2 are activated via the conserved membrane proximal-binding domain of the receptors, and JAK1 plays a major role in the signal response to IL-6 [54]. Moreover, Guschin et al. suggested that, although JAK2 was activated, it could not mediate the efficient activation of STAT1 and STAT3 after IL-6 stimulation in the absence of JAK1 [55]. Thus, JAK2 may share redundant functions with JAK1. In our model, we used two different JAK species for IFN-gamma and IL-6 receptors, respectively.

Our model contains two main components: IL-6 signalling via the JAK/STAT3 pathway and IFN-gamma signalling via the JAK/STAT1 pathway. There are multi-level interactions between the two pathways. The model contains 108 species, 192 kinetic parameters and 119 reactions, of which 103 reactions are based on previous models whereas 16 reactions were new. To make the experimental results easier to compare, if not specified otherwise, the concentrations of STAT1* and STAT3* were the sum of the concentrations of all species containing activated STAT1 and STAT3, respectively, including their monomers and dimers.

### Responses of the crosstalk model to separate IFN-gamma and IL-6 stimulation

**Figure 1:**
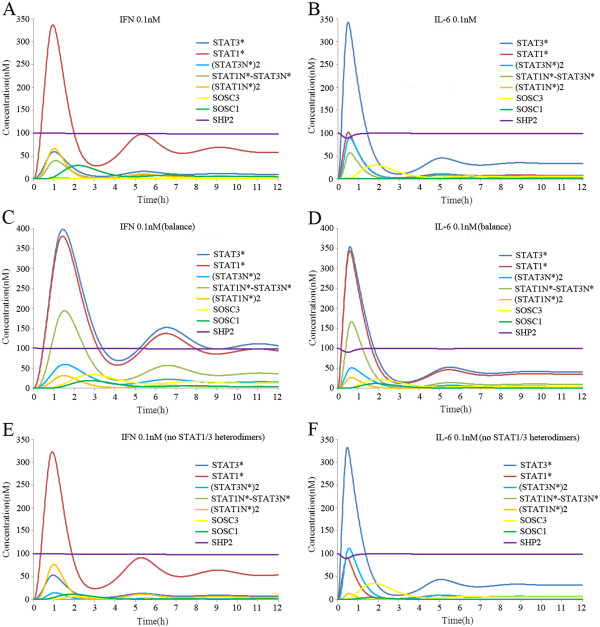
**Simulated time courses of species in the crosstalk model with continuous exposure to IFN-gamma (0.1 nM) or IL-6 (0.1 nM).** (**A**) IFN-gamma stimulation induced much stronger activation of STAT1 than STAT3. (**B**) IL-6 simulation induced much stronger activation of STAT3 than STAT1. (**C**) In a balanced competition model, IFN-gamma stimulation induced similar activation of STAT1 and STAT3. (**D**) In a balanced competition model, IL-6 stimulation induced similar activation of STAT1 and STAT3. (**E**) With no formation of STAT1/3 heterodimers, IFN-gamma stimulation induced more STAT1 and STAT3 homodimer formation than normal conditions. (**F**) With no formation of STAT1/3 heterodimers, IL-6 stimulation induced higher STAT1 and STAT3 homodimer formation than normal conditions. In the Figure, SHP2 represents the monomers of inactive SHP2, which did not combine with other molecules or complexes.

First, we stimulated the model with IFN-gamma (0.1 nM) for 12 h and found that STAT1* reached its maximum concentration (350 nM) within about 1 h, before it decreased rapidly due to the feedback inhibition of SOCS1 and SHP2. It finally reached a new steady state (60 nM) after about 6 h. STAT3 was activated to reach its maximum concentration (60 nM) within about 1h and it decreased rapidly to the control level after 2 h, whereas the activation of STAT1 was much stronger than STAT3 after IFN-gamma stimulation (see Figure 1A). The signal transduction profiles of these molecules were consistent with previous experiment results, although there were some differences in the signal strength and duration [18]. Next, we stimulated the model with IL-6 (0.1 nM) for 12 h and found that STAT3* reached its maximum concentration (350 nM) within about 0.5 h, before it decreased rapidly due to feedback inhibition from SOCS3 and SHP2. It reached a new steady state (40 nM) after about 6 h. STAT1 was activated and reached its maximum concentration (100 nM) within about 0.5 h, before it decreased rapidly to the control level after 1.5 h, whereas the activation of STAT3 was much stronger than STAT1 after IL-6 stimulation (Figure 1B). The signal transduction profiles of these molecules agreed with experiment results [20]. Next, same kinetic affinities were set for IFNR and gp130 in STAT1 and STAT3, respectively. As a result, IFN-gamma and IL-6 stimulation caused similar strong activation of STAT1, STAT3, SOCS1 and SOCS3 (Figure 1C-D). The balanced activation of STAT1 and STAT3 after IFN-gamma and IL-6 stimulation did not agree with previous experimental observations. These results demonstrated the validity of our unbalanced competition model.

**Figure 2:**
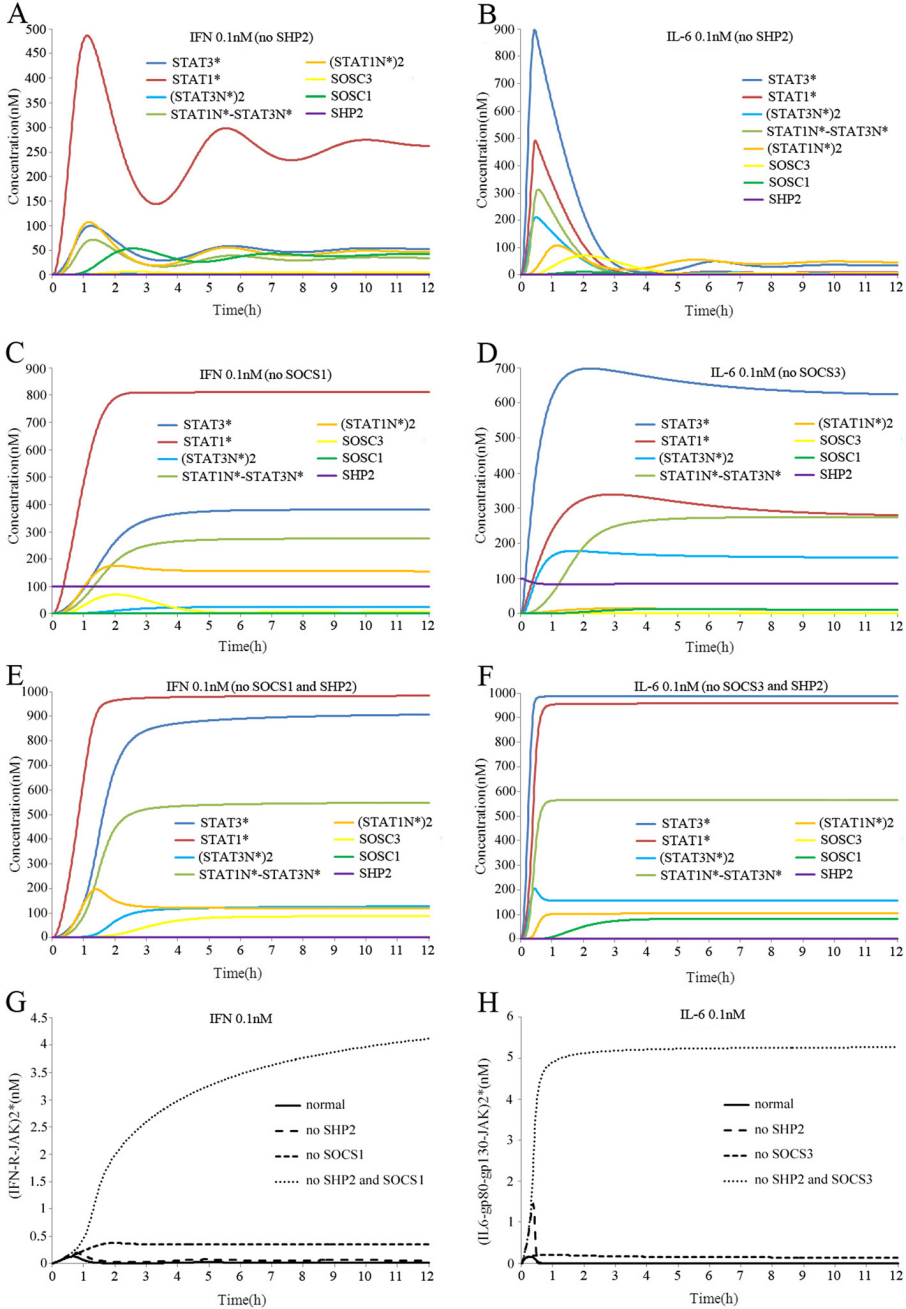
**Simulated time courses of species in the crosstalk model with continuous exposure to IFN-gamma (0.1 nM) or IL-6 (0.1 nM) after knocking out SHP2 and/or SOCSs.** (**A**) Without SHP-2, IFN-gamma stimulation induced higher levels of STAT1* and STAT3* (about 150%) than normal conditions. (**B**) Without SHP-2, IL-6 stimulation induced rapid increases in STAT1* and STAT3*. (**C**) Without SOCS1, IFN-gamma stimulation induced higher levels of STAT1* and STAT3* (about 250%) than normal conditions. (**D**) Without SOCS3, IL-6 stimulation induced rapid increases in STAT1* and STAT3*, the maximum concentrations of which reached 900 nM and 500 nM, respectively. (**E**) With SHP-2 and SOCS1 combined knockout, IFN-gamma stimulation produced a similar strength of STAT1* and STAT3*, which both rapidly reached their maximum concentrations of 960 nM and 900 nM, respectively. (**F**) With SHP-2 and SOCS3 combined knockout, IL-6 produced similar strengths of STAT1* and STAT3*, which both rapidly reached their maximum concentrations of 950 nM and 980 nM, respectively. (**G**) With SHP-2 and SOCS1 combined knockout, the level of (IFN-R-JAK)2* increased significantly after IFN-gamma stimulation. (**H**) With SHP-2 and SOCS3 combined knockout, the level of (IL6-gp80-gp130-JAK)2* increased significantly after IL-6 stimulation.

We also investigated the signal transduction profiles of STAT homo- and heterodimers in the nucleus after IFN-gamma and IL-6 stimulation, separately. After continuous stimulation with IFN-gamma (0.1 nM) for 12 h, (STAT1N*)2 reached its maximum concentration (65 nM) within about 1 h and it maintained a new steady state (5nM) after 6 h, whereas (STAT3N*)2 only reached its maximal concentration (1 nM) after 1 h (Figure 1A). By contrast, IL-6 stimulation (0.1 nM) for 12 h made the (STAT3N*)2 level reach its maximum concentration (90 nM) within about 0.5 h and it reached a new steady state (5 nM) after about 6 h, whereas (STAT1N*)2 only reached its maximal concentration (1 nM) after 0.5 h (Figure 1B). Our results confirmed the experimental observations of Haan et al. who showed that IL-6 stimulation led to STAT3 homodimers predominating in the nucleus [48]. These results suggested that IFN-gamma and IL-6 signalling preferentially activate nuclear STAT homodimers. For the STAT1/3 heterodimers (STAT1N*-STAT3N*) in the nucleus, however, both IFN-gamma and IL-6 could induce a similar concentration/strength, which reached its maximum concentration (50 nM) in about 0.5–1 h (Figure 2A–B). IFN-gamma and IL-6 could both activate STAT1 and STAT3, but fewer STAT1* and STAT3* molecules were sequestered by STAT1/3 heterodimers, so its transcriptional activation function was repressed. When the formation of STAT1/3 heterodimers was blocked, the maximum concentrations of (STAT1N)*2 and (STAT3N)*2 both increased to about 15 nM after IFN-gamma or IL-6 stimulation (Figure 1E-F). Thus, the formation of STAT1/3 heterodimers enhanced the preferential signal transduction of IFN-gamma and IL-6.

SHP-2 knockout simulations were also performed to characterize the effects of SHP-2. As shown in Figure 2A–B, knocking out SHP-2 enhanced the signal responses of IFN-gamma and IL-6, which agreed with previous experimental observations. You et al. showed that IFN-gamma could induce a higher signal response in SHP-2 null cells [14]. Schapter et al. also reported that over-expression of an inactive SHP-2 mutant in HepG2 cells enhanced STAT activation after IL-6 stimulation [15]. After IFN-gamma or IL-6 stimulation, however, the JAK/STAT pathway exhibited different features to those when knocking out SHP-2. Without SHP-2, IFN-gamma stimulation induced higher levels of STAT1* and STAT3* (about 150%) than that in normal conditions (Figure 2A). By contrast, IL-6 stimulation induced rapid increases in STAT1* and STAT3*, the maximum concentrations of which reached 900 nM and 500 nM, respectively, which was about three times higher than that in normal conditions. After IL-6 stimulation, we also observed that SOCS3 reached a peak value about 75 nM at 2 h, which inhibited signal transduction by IL-6 and quickly caused the concentrations of STAT1* and STAT3* to drop to normal levels after 3 h (Figure 2B). Knockout simulations were also performed for SOCS1 and SOCS3. As shown in Figure 2C–D, knocking out SOCS1 enhanced the activation of STAT1 after IFN-gamma stimulation, while knocking out SOCS3 enhanced the activation of STAT3 after IL-6 stimulation. Our simulation results agreed with previous experimental observations. Brysha et al. demonstrated that in vitro and in vivo hepatocytes lacking SOCS-1 exhibited prolonged activation of STAT1 after IFN-gamma stimulation, which correlated with the dramatically increased sensitivity to the toxic effects of IFN-gamma [13]. Niwa et al. reported that inhibition of SOCS3 expression enhanced the activation of STAT3 and cell growth [56]. After IFN-gamma or IL-6 stimulation, however, the JAK/STAT pathway exhibited different features when knocking out SOCS1 or SOCS3. Without SOCS1, IFN-gamma stimulation induced higher levels of STAT1* and STAT3* (about 250%) compared with those in normal conditions (Figure 2C). Without SOCS3, however, IL-6 stimulation induced increases in STAT1* and STAT3*, the maximum concentrations of which reached 700 nM and 300 nM, respectively, which were about double those in normal conditions. After IL-6 stimulation, we also observed that SHP-2 dropped to a low level of about 80 nM at 1 h, which attenuated signal transduction by IL-6 and caused the concentrations of STAT1* and STAT3* to fall slowly after 3 h (Figure 2D). Thus, SOCS1 and SOCS3 had different interaction patterns with SHP-2. SOCS1 and SHP-2 synergistically regulated signal transduction by IFN-gamma. Knocking out SOCS1 or SHP-2 enhanced the integral activation of STAT1 induced by IFN-gamma stimulation. By contrast, SOCS3 and SHP-2 regulated signal transduction by IL-6 in a more complementary manner. Knocking out SHP-2 alone enhanced the rapid response of the IL-6 signal, due to a compensatory increase in SOCS3. Knocking out SOCS3 also led to lower levels of SHP-2, which caused a slow decline in STAT1* and STAT3* 3 h after IL-6 stimulation. Simulations of the combined knockout of SHP-2 and SOCS were performed to characterize their joint effects on IFN-gamma and IL-6 stimulations. First, we stimulated the SHP-2 and SOCS1 combined knockout model with IFN-gamma (0.1 nM) for 12 h and found that STAT1* reached its maximum concentration (960 nM) within about 2 h while STAT3* reached its maximum concentration (900 nM) within about 3 h (Figure 2E). IFN-gamma stimulation induced a similar strength of STAT1* and STAT3* in SHP-2 and SOCS1 combined knockout conditions. We then stimulated the SHP-2 and SOCS3 combined knockout model using IL-6 (0.1 nM) for 12 h and found that STAT1* and STAT3* both quickly reached their maximal concentration of 950 nM and 980 nM, respectively, within about 1 h (Figure 2F). IL-6 stimulation also induced similar strengths of STAT1* and STAT3* in SHP-2 and SOCS3 combined knockout conditions. Thus, the combined knockout of SHP-2 and SOCSs abolished the preferential activation of IFN-gamma and IL-6. The unbalance competition between STAT1 and STAT3 was not related directly to SHP-2 and SOCSs, but SHP-2 and SOCSs combined with the activated receptor complexes and inhibited signal transduction via the JAK/STAT pathway [2,6,12]. Therefore, we deduced that SHP-2 and SOCSs could limit the concentration of active receptor complexes, which indirectly affected the preferential activation of IFN-gamma and IL-6. Therefore, we investigated the signal transduction profiles of the activated receptor complexes in response to IFN-gamma and IL-6 with and without knocking out SHP-2 and/or SOCSs. Without any knockout, (IFN-R-JAK)2* reached its maximum concentration (0.1 nM) in about 0.5 h after IFN-gamma, before decreasing rapidly. After knocking out SHP-2, the level of (IFN-R-JAK)2* increased by about 120% compared with that in normal conditions. Without SOCS1, (IFN-R-JAK)2* increased rapidly and reached a new steady state (0.35 nM) after 2 h, whereas the combined knockout of SHP-2 and SOCS1 caused the level of (IFN-R-JAK)2* to increase significantly, reaching 4.5 nM in 12 h, which was about forty time as high as that in normal conditions (Figure 2G). Without any knockout and with IL-6, (IL6-gp80-gp130-JAK)2* reached its maximum concentration (0.15 nM) within about 0.25 h, before decreasing rapidly. After knocking out SHP-2, (IL6-gp80-gp130-JAK)2* increased rapidly and it reached its maximum concentration of 1.4 nM in 0.25 h, which was about nine times that in normal conditions, although it quickly returned to a normal level after 0.5 h. With SOCS3 knock out, the (IL6-gp80-gp130-JAK)2* level increased and reached a new steady state (0.15 nM) after 1 h. With the combined knockout of SHP-2 and SOCS3, the levels of (IL6-gp80-gp130-JAK)2* increased significantly and reached a new steady state (5 nM) after 1 h, which was about 35 times that in normal conditions (Figure 2H). The simulation results demonstrated that with the SHP-2 and SOCSs combined knockout, the levels of (IFN-R-JAK)2* and (IL6-gp80-gp130-JAK)2* increased significantly after IFN-gamma and IL-6 stimulation. STAT1 and STAT3 competed for the same motifs in IFNR and gp130, but there was sufficient (IFN-R-JAK)2* and (IL6-gp80-gp130-JAK)2*, so the preferential activations of IFN-gamma and IL-6 were abolished. These simulated observations still await further experimental verification.

### Responses of the crosstalk model after disrupting STAT1 and STAT3

**Figure 3:**
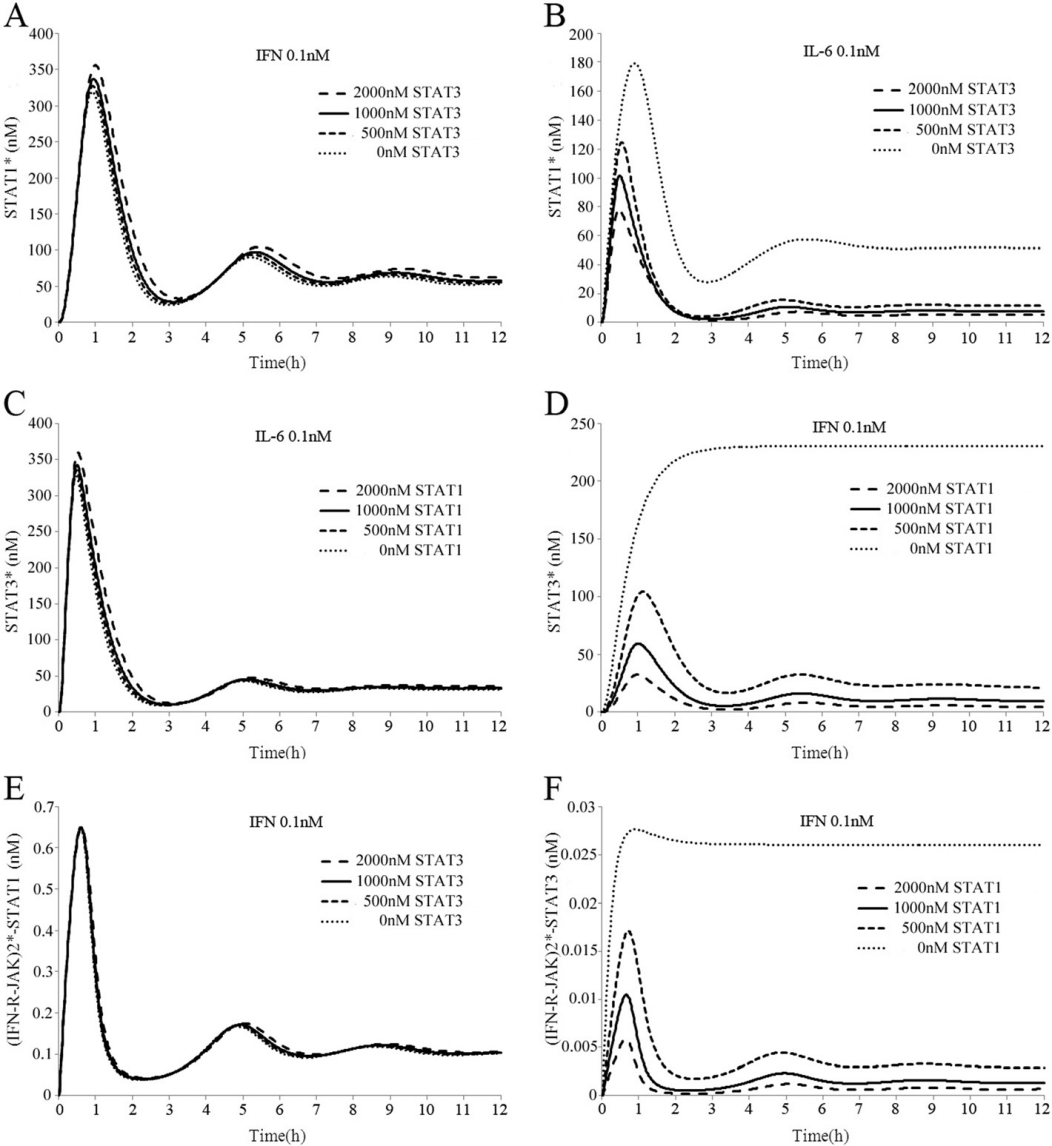
**Dependency of JAK/STAT pathway activation on the initial concentrations of STATs.** (**A**) The activation of STAT1 was mainly independent of the concentration of STAT3 after IFN-gamma simulation. (**B**) The concentration of STAT3 significantly affected the activation of STAT1 after IL-6 simulation. (**C**) The activation of STAT3 was mainly independent of the concentration of STAT1 after IL-6 simulation. (**D**) The concentration of STAT1 significantly affected the activation of STAT3 after IFN-gamma simulation. (**E**) Recruitment from STAT1 to (IFN-R-JAK)2* was mainly independent of the concentration of STAT3 after IFN-gamma simulation. (**F**) The concentration of STAT1 significantly affected recruitment from STAT3 to (IFN-R-JAK)2*.

The effect of STAT3 on signal transduction via the JAK/STAT pathway was analyzed by varying the initial concentration of STAT3 in a range of 0–2000 nM. We found that changing the STAT3 level did not significantly affect the state of STAT1* after IFN-gamma stimulation (Figure 3A), which was consistent with previous experimental observations [22]. By contrast, the level of STAT1* was clearly affected by the initial STAT3 concentration in response to IL-6. In particular, when STAT3 was knocked out, STAT1 was more phosphorylated and for longer, so STAT1* reached its maximum concentration (180 nM) in about 1 h, which was about double that in normal conditions. Finally, it reached a new steady state (50 nM) after about 7 h (Figure 3B). This was consistent with previous experimental results, although there were some differences in the signal strength and duration [20]. The different signal responses to IFN-gamma and IL-6 during STAT3 disruption may explain why IL-6, but not IFN-gamma, could trigger apoptosis and inhibit the in vivo growth of human malignant T cells after knocking out STAT3 [22]. Next, we analyzed the effect of STAT1 on signal transduction via the JAK/STAT pathway by varying the initial concentration of STAT1 in a range of 0–2000nM. We found that changing the initial concentration of STAT1 did not significantly affect the level of STAT3* after IL-6 stimulation (Figure 3C). By contrast, the level of STAT1 dramatically affected the status of STAT3* after IFN-gamma stimulation. When we knocked out STAT1 in our model, IFN-gamma stimulation also led to much stronger activation of STAT3, which caused a significant increase in the levels of STAT3*. It finally reached a new steady state (220 nM) after 1 h, which was about three times that in normal conditions (Figure 3D). Our simulation results were consistent with previous experimental observations [19]. Therefore, IFN-gamma and IL-6 signalling could mutually switch in the conditions of STAT1 or STAT3 knockout, which agreed very well with previous experimental observations [19,20].

After IFN-gamma and IL-6 stimulation, the recruitment of STAT1 and STAT3 to the activated receptor complexes directly affected their phosphorylation, which has important roles in signal transduction by IFN-gamma and IL-6 [34-36]. In our unbalanced competition model, STAT1 and STAT3 had different affinities for IFNR and gp130, so we deduced that disrupting STAT1 and STAT3 may have different effects on the recruitment of STAT1 and STAT3. Next, we investigated the effect of changing the initial concentration of STAT1 and STAT3 on the associations of STATs with activated receptor complexes in response to IFN-gamma and IL-6. Our simulation results demonstrated that changing the concentration of STAT3 had little effect on the formation of (IFN-R-JAK)2*-STAT1 (Figure 3E), whereas altering the STAT1 level significantly affected the formation of (IFN-R-JAK)2*-STAT3 (Figure 3F) after IFN-gamma stimulation. Our simulations also showed that the formation of (IL6-gp80-gp130-JAK)2*-STAT3 was almost independent of STAT1 disruption after IL-6 stimulation, although changing the STAT3 level significantly affected the formation of (IL6-gp80-gp130-JAK)2*-STAT1(data not shown). Therefore, the unbalanced competition between STAT1 and STAT3 for IFNR and gp130 was not only the pivotal mechanism for the preferential activation of IFN-gamma and IL-6, but it also determined the recruitment of STAT1 and STAT3 to the activated receptor complexes.

### Responses of the crosstalk model to combined stimulation with IFN-gamma and IL-6

**Figure 4:**
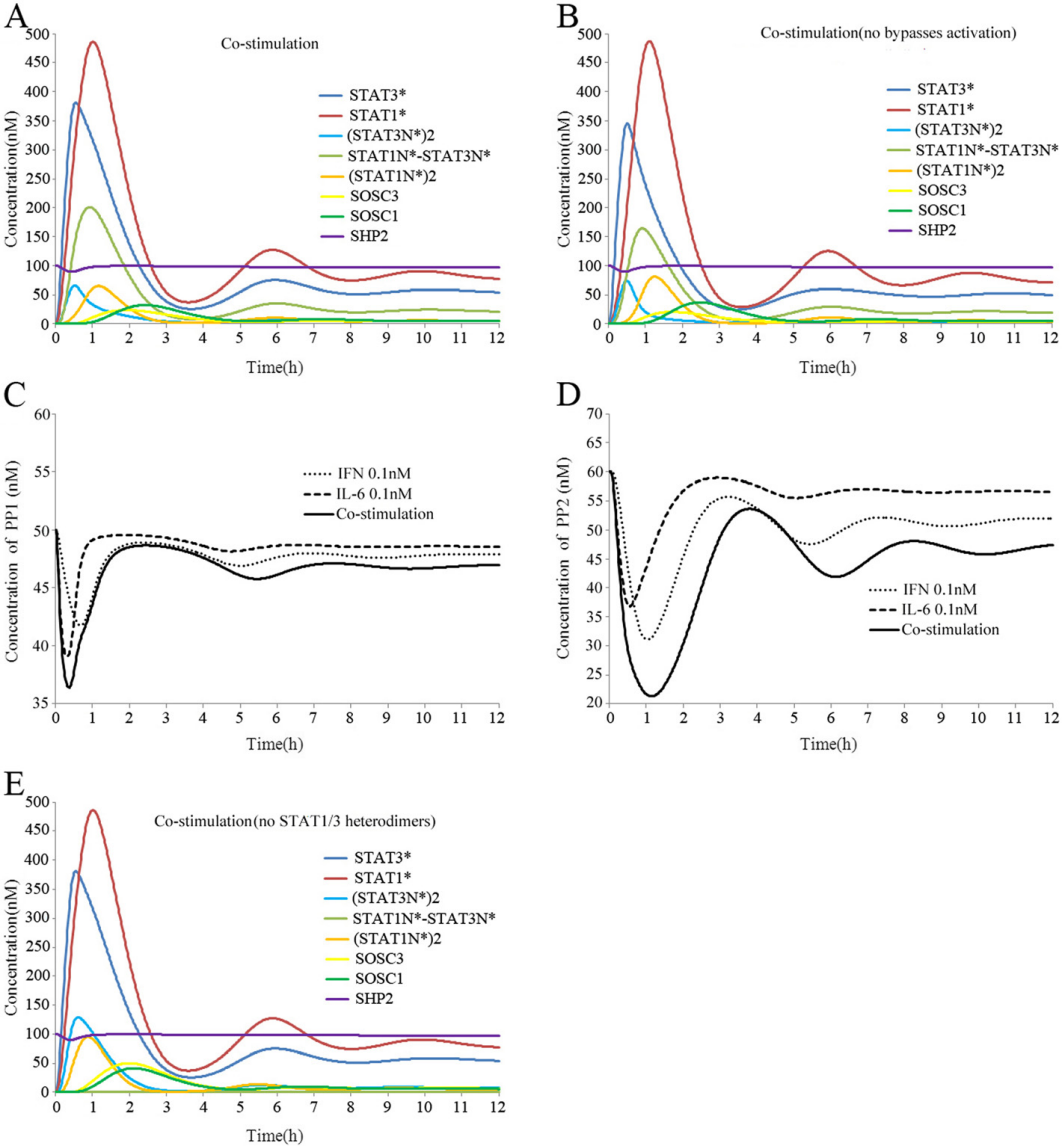
**Simulated time courses of species in the crosstalk model after combined stimulation with IFN-gamma and IL-6.** (**A**) Combined stimulation with IFN-gamma and IL-6 induced higher activation of STAT1 and STAT3 than separate treatments. (**B**) A setting where STAT1 could only be activated by IFN-gamma and STAT3 could only be activated by IL-6 did not abolish the higher activation of STAT1 and STAT3 compared with individual stimulation experiment. (**C**) Combined stimulation with IFN-gamma and IL-6 reduced the level of PP1 compared with individual treatments. (**D**) Combined stimulation with IFN-gamma and IL-6 reduced the level of PP2 compared with the individual treatments. (**E**) Without the formation of STAT1/3 heterodimers, combined stimulation with IFN-gamma and IL-6 did not induce higher levels of (STAT1N*)2 and (STAT3N*) compared with normal conditions.

We considered a combined stimulation protocol where the model was stimulated with IFN-gamma (0.1 nM) and IL-6 (0.1 nM) together for 12 h. Figure 4A shows that compared with the separate treatments, the combined stimulation induced higher activation of the JAK/STAT pathway. The dynamic responses of the JAK/STAT pathway were consistent with previous results reported in the literature [18]. In the previous section, we showed that IFN-gamma and IL-6 could activate both STAT1 and STAT3, which may explain the higher activation of STAT1 and STAT3 after combined stimulation. However, when STAT1 could only be activated by IFN-gamma and STAT3 could only be activated by IL-6, combined stimulation still caused greater activation of STAT1 and STAT3 than separate stimulation (Figure 4B). Thus, we inferred that other mechanism may play important roles in this phenomenon. PP1 and PP2 are two different types of phosphatases that dephosphorylate STAT*s in the cytoplasm and nuclei, respectively [16,17]. In our model, the total amounts of both PP1 and PP2 were fixed and only the monomers could combine and dephosphorylate the newly generated STAT*s. We then investigated the signal transduction profiles of PP1 and PP2 in response to IFN-gamma and/or IL-6 stimulation, and we found that combined stimulation with IFN-gamma and IL-6 could activate STAT1 and STAT3, which resulted in lower levels of PP1 and PP2 than the separate treatments (Figure 4C–D). After combined stimulation, the activation of STAT1 prevented STAT3 from being dephosphorylated and vice versa. This mechanism contributed to the higher activation of STAT1 and STAT3 after combined stimulation with IFN-gamma and IL-6.

Signal transduction via the JAK/STAT pathway depended on the formation of STAT homodimers [10], which are regarded as the main transcription factors during IFN-gamma and IL-6 signalling [2,8]. We further investigated whether combined stimulation with IFN-gamma and IL-6 could induce higher (STAT1N)*2 and (STAT3N)*2 than separate treatments. Figure 4A shows that 12 h after combined stimulation, the STAT homodimers were not induced at a higher level than the separate treatments. However, STAT1N*-STAT3N* reached their maximum concentration (200 nM) within about 1 h, which was about 3 time higher than the individual treatment. The formation of STAT1N*-STAT3N* greatly restricted the formation of STATs homodimers. After we abolished the formation of STAT1/3 heterodimers, the maximum concentrations of (STAT3N)*2 and (STAT1N)*2 increased to about 100 nM with combined stimulation (Figure 4E). Combined stimulation with IFN-gamma and IL-6 led to greater activation of both STAT1 and STAT3, but the formation of STAT1/3 heterodimers played an important role in preventing mutual strengths between IFN-gamma and IL-6 signalling.

### Responses of the crosstalk model to successive IFN-gamma and IL-6 stimulation

We analyzed previous studies that focused on the interactions between IFN-gamma and IL-6 signalling and found that their interactions were asymmetric. Bluyssen et al. reported that pre-treatment of EC with IFN-gamma significantly decreased STAT3* induction by IL-6 without affecting the total amount of STAT3 [18]. By contrast, Kaur et al. reported that STAT1 activation induced by IFN-gamma was mainly unchanged after pre-treatment IL-6 or other gp130-related cytokines in SH-SY5Y human neuroblastoma cells [24]. We tried to provide a reasonable explanation for the asymmetric interactions between IFN-gamma and IL-6 using simulation experiments with our model.

**Figure 5:**
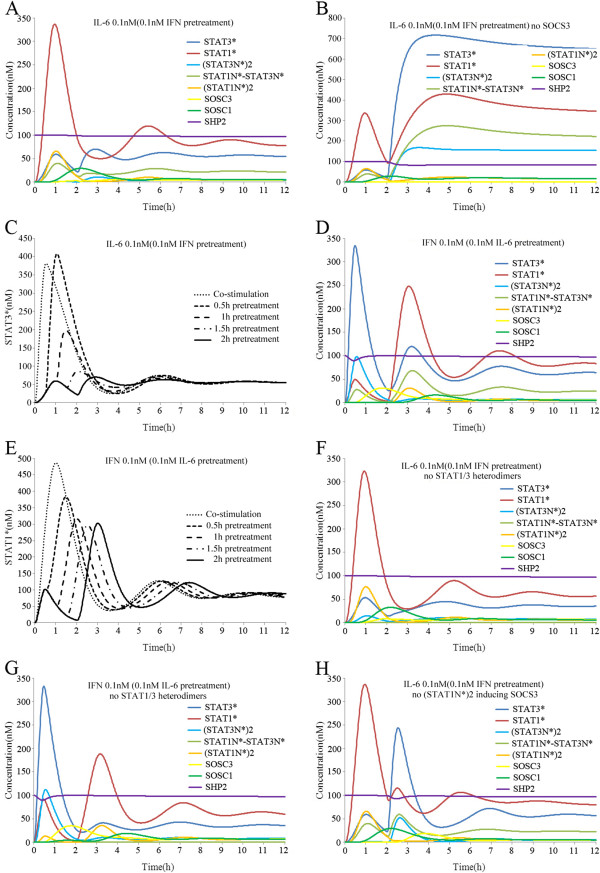
**Simulated time course of species in the crosstalk model of IL-6 after pre-treatment with IFN-gamma or IFN-gamma after pre-treatment with IL-6.** (**A**) Pre-treatment with IFN-gamma significantly decreased the activation of STAT3 induced by IL-6. (**B**) Without SOCS3, the inhibitory effect of IFN-gamma on STAT3* induction by IL-6 was completely eliminated. (**C**) Temporal pre-treatment with IFN-gamma partly inhibited IL-6 signalling. (**D**) Pre-treatment with IL-6 reduced the amount of STAT1* only slightly and did not inhibit the response of IFN-gamma. (**E**) Changing the length of pre-treatment with IL-6 did not affect the signal response of IFN-gamma. (**F**) Without the formation of STAT1/3 heterodimers, IL-6 had a higher capacity to inhibit IFN-gamma signal transduction. (**G**) Without the formation of STAT1/3 heterodimers, IFN-gamma had a higher capacity to inhibit IL-6 signal transduction. (**H**) Abolishing (STAT1N)*2 induction of SOCS3 inhibited the IFN-gamma effects on IL-6.

First, we stimulated the model with IFN-gamma (0.1 nM) for 12 h, which we started 2 h prior to IL-6 (0.1 nM) stimulation. IL-6 slightly increases the level of STAT3*, but pre-treatment with IFN-gamma significantly decreased STAT3* induction by IL-6 (Figure 5A). This was consistent with the results reported by Bluyssen et al. [18]. SOCS3 is a negative regulator of IL-6 signalling and it can be induced by IFN-gamma stimulation, so we deduced that SOCS3 may have an important role during inhibition. When we knocked out SOCS3, the inhibitory effect of IFN-gamma on STAT3* induction by IL-6 was eliminated completely (Figure 5B). These results showed that SOCS3 was an essential component in the inhibition of IFN-gamma to IL-6 signalling. We also found that the expression of SOCS3 had a time delayed feedback, which significantly increased 1 h after IFN-gamma stimulation. Therefore, we deduced that temporal pre-treatment with IFN-gamma may not have induced sufficient SOCS3 to inhibit IL-6 signalling. Figure 5C shows that temporal pre-treatment with IFN-gamma partly inhibited IL-6 signalling and that the duration of pre-treatment with IFN-gamma needed to be longer than 1 h to achieve this inhibition. We then investigated how pre-treatment with IL-6 affected the IFN-gamma signal response. Our simulation results showed that pre-treatment with IL-6 for 2 h only slightly reduced the amount of STAT1* and did not inhibit the signal response of IFN-gamma (Figure 5D), while changing the duration of the pre-treatment with IL-6 still had no obvious effect on the signal response of IFN-gamma. Moreover, pre-treatment for less than 1 h had almost no effect on the state of STAT1* (Figure 5E). These simulation results were consistent with the results reported by Kaur et al. [24]. We inferred that the asymmetric interactions between IFN-gamma and IL-6 signalling were related mainly to the different inhibition efficiencies of SOCS1 and SOCS3. SOCS1 could be induced by (STAT1N)*2 after IL-6 stimulation, but SOCS1 induction by IL-6 is not sufficient to inhibit IFN-gamma signalling. After IFN-gamma stimulation, however, SOCS3 could be induced by (STAT1N)*2 and (STAT3N)*2, which achieved the inhibition from IFN-gamma to IL-6.

The formation of STAT1/3 heterodimers also contributed to the asymmetric interactions between IFN-gamma and IL-6 signalling. As previously described, the formation of STAT1/3 heterodimers enhanced the preferential signal transduction of IFN-gamma and IL-6 by sequestering a fraction of STAT1* and STAT3*. After we abolished the formation of STAT1/3 heterodimers, the maximum concentration of SOCS1 induced by IL-6 increased to 4.2 nM within about 1.5 h, and IL-6 exhibited a greater capacity for inhibiting IFN-gamma signalling (Figure 5F). Abolishing the formation of STAT1/3 heterodimers also enhanced the inhibition from IFN-gamma to IL-6 (Figure 5G). In addition, the mechanism of (STAT1N*)2 inducing SOCS3 also played an important role in the asymmetric interactions. The concentration of (STAT3N*)2 induced by IFN-gamma stimulation was very low due to the sequestering effect of STAT1/3 heterodimers. Therefore, we deduced that SOCS3 induction by (STAT3N*)2 was not sufficient to achieve the inhibition from IFN-gamma to IL-6. Indeed, when we abolished the (STAT1N)*2 induction of SOCS3, the inhibition from IFN-gamma to IL-6 was clearly mitigated (Figure 5H). IFN-gamma only slightly reduced the activation of STAT3 induction by IL-6, which did not agree with previous experimental observations [18]. Therefore, (STAT1N*)2 induction of SOCS3 was essential for the inhibition from IFN-gamma to IL-6 during signal transduction.

### Argument for the non-competitive model

Ho et al. reported that STAT3 did not affect the activation of STAT1 during type I interferon responses [23]. They argued that STAT3 did not suppress STAT1 activation via tyrosine phosporylation and they excluded inhibitory mechanisms such as the competition for docking sites and the inhibition of signalling events upstream of STAT1 tyrosine phosphorylation. Based on their argument, we proposed an alternative hypothesis for the combined pattern between STATs and the receptors for IFN-gamma and IL-6 in addition to our previous competition model, which we refer to as the non-competitive model. In the non-competitive model, STAT1 and STAT3 do not compete for the same docking sites of IFNR and gp130, so we considered that STAT1 and STAT3 could bind different phosphorylated docking sites in IFNR and gp130 via their SH2 domain. Based on these considerations, we added the new biochemical reactions (N17) - (N20) (see “non-competitive model” in Additional file 1) to simulate the mutually independent combination of STAT1 and STAT3 with the receptor complexes. STAT3 did not suppress the activation of STAT1 via tyrosine phosphorylation, so we considered that the combination of STAT3 with receptor complexes did not suppress the phosphorylation processes of STAT1 and vice versa. Based on these considerations, we added the new biochemical reactions (N21) - (N24) to simulate the mutually independent phosphorylation of STAT1 and STAT3 within receptor complexes. In addition, we estimated the parameters for reactions (N17)-(N24) and re-estimated the parameters for reactions (N1)-(N6) to fit the non-competitive model (Additional file 1: Table S5). Detailed descriptions of the non-competitive model written in COPSI (using the filename extension “.cps”) are available in Additional file 2.

**Figure 6:**
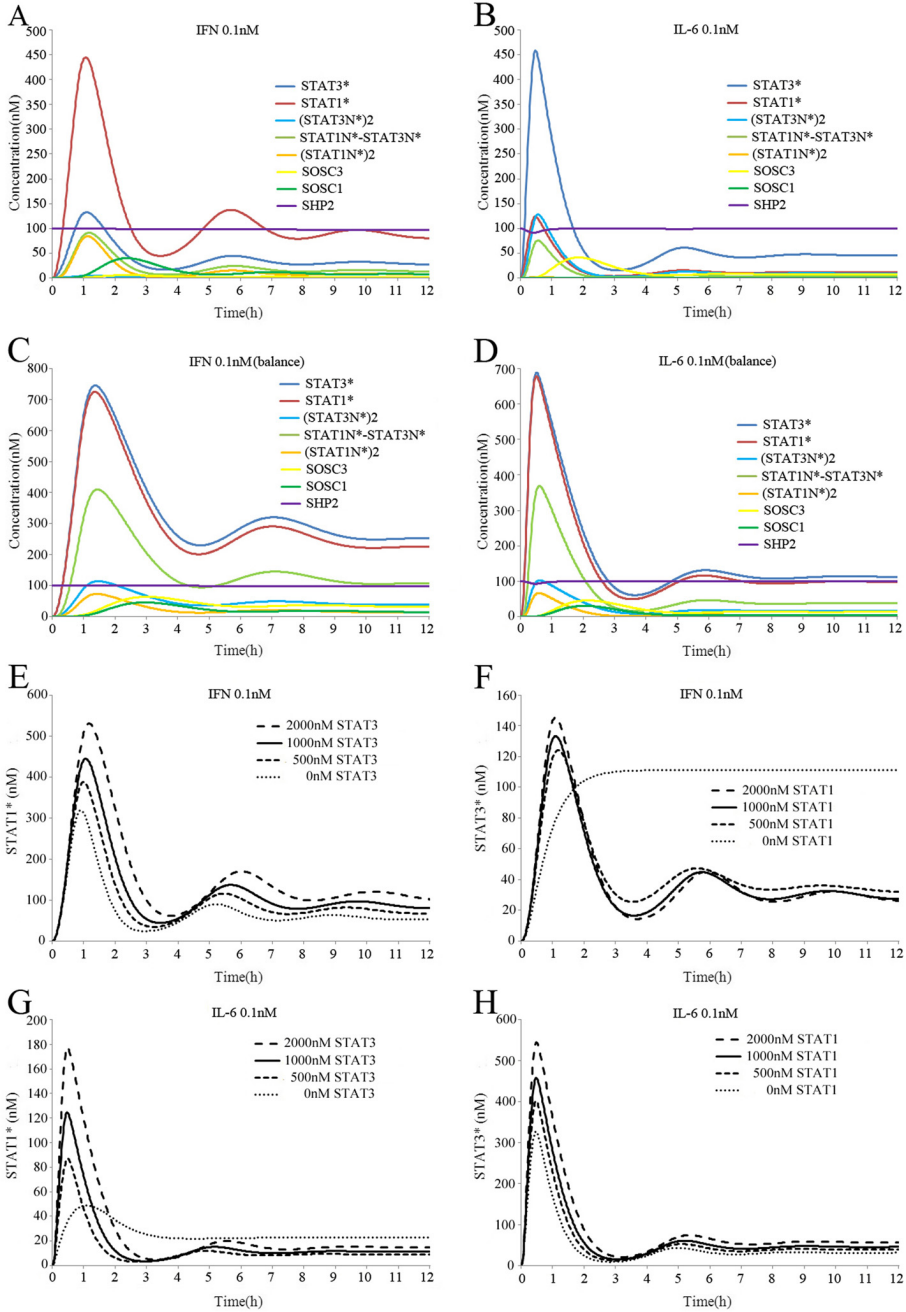
**Simulated time courses of species in the non-competitive model with continuous exposure to IFNγ (0.1 nM) or IL-6 (0.1 nM).** (**A**) IFNγ stimulation induced much stronger activation of STAT1 than STAT3. (**B**) IL-6 simulation induced much stronger activation of STAT3 than STAT1. (**C**) With balanced kinetic affinities, IFNγ stimulation induced similar activation of STAT1 and STAT3. (**D**) With balanced kinetic affinities, IL-6 stimulation induced similar activation of STAT1 and STAT3. (**E**) STAT1 was phosphorylated more strongly and for longer with a higher level of STAT3 after IFNγ simulation. (**F**) STAT3 was phosphorylated more strongly and for longer with a higher level of STAT1 after IL-6 simulation.

First, we conducted simulation experiments using IFN-gamma and IL-6 stimulation separately, as in the previous study, and we aimed to estimate and optimize the parameters of the new non-competitive model. As shown in Figure 6A-B, our computational simulation indicated that the activation of STAT1 was much greater than STAT3 after IFN-gamma stimulation, whereas the activation of STAT3 was much greater than STAT1 after IL-6 stimulation, which agreed with previous experiment results despite some deviations in the signal strength and durations [19,21]. Next, the same kinetic affinities were used for IFNR and gp130 with STAT1 and STAT3, respectively. IFN-gamma and IL-6 stimulation caused similar strong activation of STAT1, STAT3, SOCS1 and SOCS3 (Figure 6C-D), which did not agree with previous experimental observations. These results demonstrated that the non-competitive model using our estimated parameter set could satisfy the basic simulation requirements and that it simulated the preferential activation of IFN-gamma and IL-6.

Next, we executed the same series simulation experiments described in the previous sections using the non-competitive model. We found that the non-competitive model approximately simulated the crosstalk between IFN-gamma and IL-6 signalling in all conditions (data not shown), with the exception of the disrupted STAT1 and STAT3 conditions. Figure 6E shows that when we fixed the concentration of STAT1 at 1000 nM and changed the concentration of STAT3 from 0, 500, 1000, to 2000 nM, the states of STAT1* were obviously dependent on the level of STAT3 after IFN-gamma stimulation. In particular, STAT1 was more strongly phosphorylated, and for longer, with higher levels of STAT3, which were completely different from the experimental observations reported by Regis et al. [22]. Similarly, when we fixed the concentration of STAT3 at 1000 nM and changed the concentration of STAT1 from 0, 500, 1000, to 2000 nM, the states of STAT3* were also obviously dependent on the level of STAT1 after IL-6 stimulation (Figure 6F). Moreover, STAT3 was also more strongly phosphorylated and for longer with higher levels of STAT1, which did not agree with the experimental observations of Dimberg et al. [57]. Indeed, the activation of STATs was determined by the swing between phosphorylation and dephosphorylation processes [6]. In the non-competitive model, STAT1 and STAT3 did not suppress the phosphorylation of each other, although they share the phosphatases PP1 and PP2, so the activation of STAT1 prevented STAT3 from being dephosphorylated and vice versa. The disruption of the dynamic balance between the phosphorylation and dephosphorylation of STAT1 and STAT3 meant the responses of the non-competitive model were hypersensitive to the initial concentrations of STAT1 and STAT3. Therefore, the non-competitive model may not reflect the crosstalk mechanisms between IFN-gamma and IL-6 signalling in a physiological environment. Thus, alternative hypotheses should be proposed based on new experimental observations. Here, we provide an integrated platform that facilitates the verification of possible hypotheses related to the crosstalk between IFN-gamma and IL-6 signalling in future work.

### Sensitivity analysis of the competition model

In this study, we used computational simulation to demonstrate the effectiveness of the competition model. Next, we determined the dynamic characteristics of our model by sensitivity analysis. The calculations used in the sensitivity analysis are shown in the “Methods” section. The upstream key regulatory molecules of (STAT1N*)2 and (STAT3N*)2 comprise cytokines, receptors, JAK, STAT1C, STAT3C, PP1, PP2, and SHP2, and their initial concentrations were considered in the sensitivity analysis. First, we applied a sensitivity analysis to determine the relative sensitivity of these components using IFN-gamma stimulation as the input.

**Figure 7:**
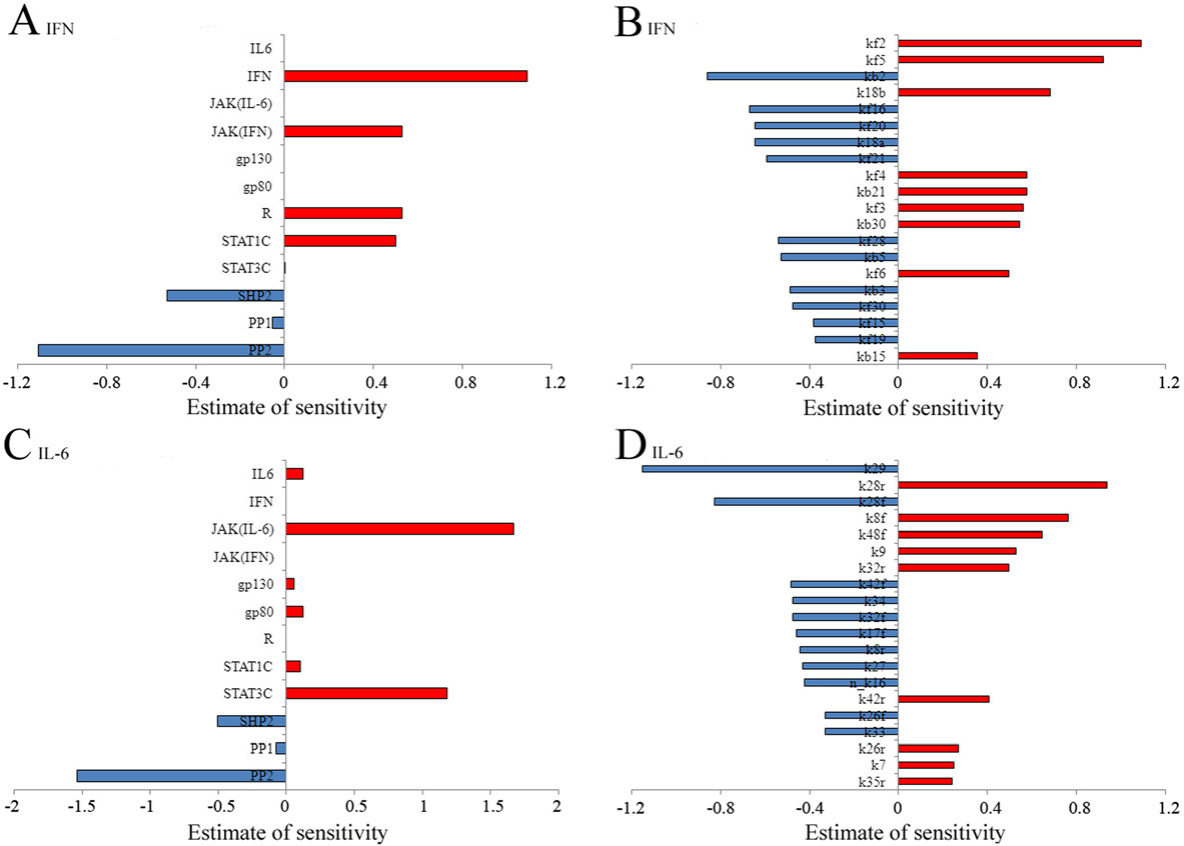
**Sensitivity analysis of the competition model.** (**A, B**) indicate the sensitivities of the initial molecule concentrations and the reaction rate constants after IFN-gamma stimulation. (**C, D**) indicate the sensitivities of the initial molecule concentrations and the reaction rate constants after IL-6 simulation. The definition of the parameter sensitivity can refer to the “Methods” section. The positive (or negative) sensitivity values indicate consistent (or opposite) percentage changes in output to the perturbation in the parameter compared to the nominal solution. The red and blue bars represent positive and negative sensitivity values, respectively. For brevity, we only show the top 20 parameters with high sensitivity values.

The results indicated that IFN-gamma, the receptor, JAK, STAT1C, and PP2 were relatively important components of the IFN-gamma-induced JAK/STAT signalling pathway (Figure 7A). It was also noted that some of the initial concentrations, such as those of PP1 and STAT3C, had little impact on the time course of (STAT1N*)2. In a previous simulation, we found that the decline in the concentration of PP2 was more obvious than that of PP1 during IFN-gamma and IL-6 signal transduction (Figure 4C–D). We deduced that the basic level of PP1 exceeded the demand of IFN-gamma transduction, which agreed with the conclusions of the previous study. The concentration of STAT3 did not affect the activation of STAT1, as described in the previous section. Moreover, we found that the parameters related to the key signalling components identified had relatively significant effects on the time course of (STAT1N*)2, as shown in Figure 7B. The reactions with the most sensitive kinetic parameters were STAT1C phosphorylation by the receptor complex of IFN-gamma and PP2 negative regulation. The SOCS1 synthesis, degradation, and negative regulation processes were also very sensitive, which confirmed the important role of the negative feedback factor SOCS1 during IFN-gamma signal transduction.

Next, we performed a sensitivity analysis using IL-6 stimulation as the input. The sensitivities of the initial concentrations and the critical kinetic parameters are shown in Figure 7C and Figure 7D. The results indicated that the reactions with PP2 negative regulation and JAK combination were highly sensitive to IL-6 stimulation. As described in previous sections, the concentration of STAT1 did not significantly affect the activation of STAT3 after IL-6 stimulation, which was also confirmed by the sensitivity analysis. Similarly, we found that the SOCS3 synthesis, degradation, and negative regulation processes were highly sensitive to IL-6 stimulation, which confirmed the important role of the negative feedback factor SOCS3 during IL-6signal transduction. In particular, we found that the phosphorylation of STAT3 in STAT1/3 heterdimers in the nucleus also had a high impact on the state of (STAT3N*)2. Overall, the sensitivity analysis determined the sensitive components and parameters during JAK/STAT signal transduction. These results provide valuable information that may inform further investigations of regulation and drug target identification in aberrant pathways. The detailed results of the sensitivity analysis of the competition model are shown in Additional file 1: Tables S6–S7.

### Sensitivity analysis of the non-competitive model

**Figure 8:**
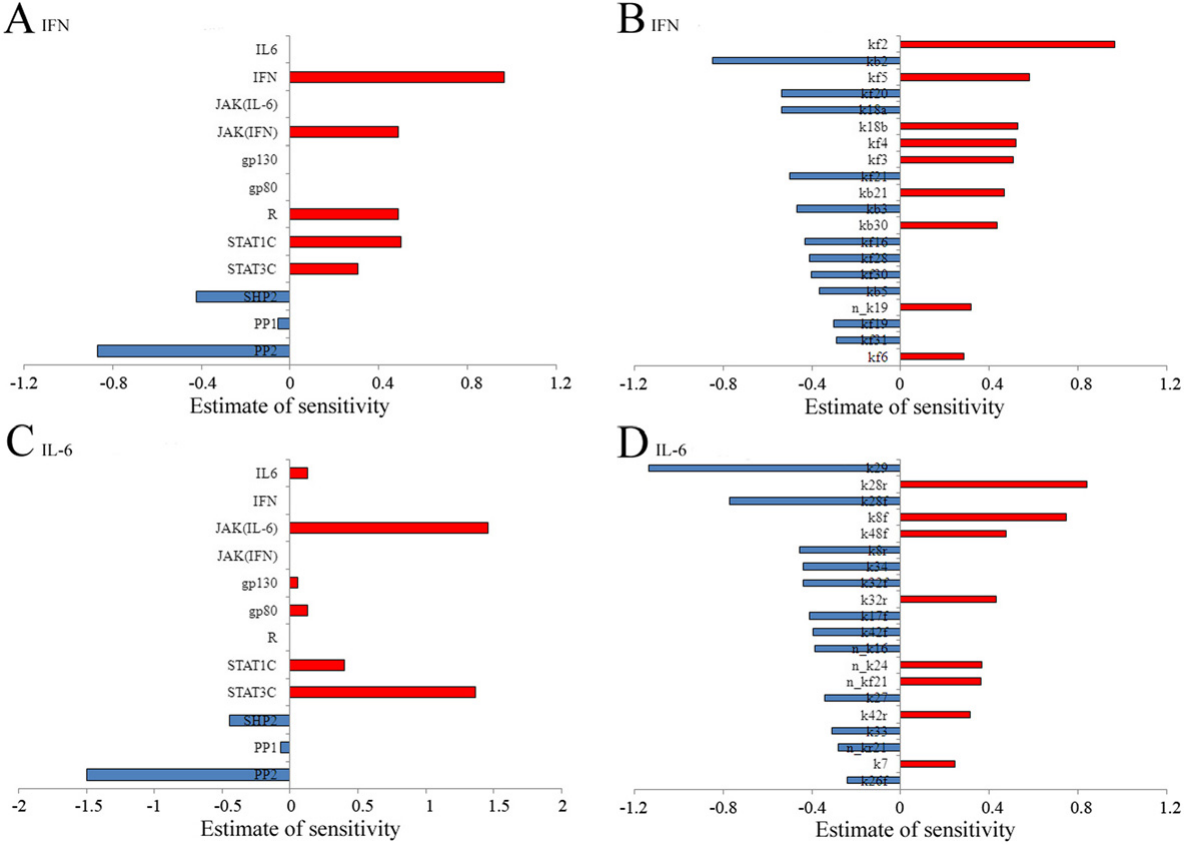
**Sensitivity analysis of the non-competitive model.** (**A, B**) indicate the sensitivities of the initial molecule concentrations and the reaction rate constants after IFN-gamma stimulation. (**C, D**) indicate the sensitivities of the initial molecule concentrations and the reaction rate constants after IL-6 simulation, respectively. The definition of the parameter sensitivity can refer to the “Methods” section. The positive (or negative) sensitivity values indicate consistent (or opposite) percentage changes in output to the perturbation in the parameter compared to the nominal solution. The red and blue bars represent positive and negative sensitivity values, respectively. For brevity, we only show the top 20 parameters with high sensitivity values.

We also investigated the dynamic characteristic of the non-competitive model by sensitivity analysis. First, we applied sensitivity analysis to determine the critical components with dominant effects where IFN-gamma stimulation was used as the input. Figure 8A shows the sensitivities to the initial concentrations, while the sensitivities of the critical kinetic parameters are shown in Figure 8B. There were some deviations in magnitude but the non-competitive model yielded similar sensitivity results to the competition model after IFN-gamma stimulation, except the concentration of STAT3C had a higher sensitivity to (STAT1N*)2 in the non-competitive model. This confirmed our previous conclusion that the responses of IFN-gamma in the non-competitive model were hypersensitive to the initial concentrations of STAT3. Next, we performed a sensitivity analysis using IL-6 stimulation as the input. Figure 8C shows the sensitivities to the initial concentrations, while the sensitivities to the critical kinetic parameters are shown in Figure 8D. We also found that the concentration of STAT1C was highly sensitive to (STAT3N*)2 in the non-competitive model. In the previous section, simulation results demonstrated the non-competitive model could not accurately reflect the signal transduction of IL-6 and IFN-gamma under the condition of disrupting of STATs. Here, the sensitivity analysis confirmed the high sensitivity of STATs in the non-competitive model. The detailed results of the sensitivity analysis of the non-competitive model are shown in Additional file 1: Tables S8–S9.

## Discussion

The results were affected by the limitations of our model. It is important to consider these limitations because they form the basis for future improvements. First, we only considered three possible levels of crosstalk between IFNγ and IL-6 systems. Haan et al. showed that, after IL-6 stimulation, STAT1 phosphorylation was entirely dependent on JAK1 kinase activity whereas STAT3 activation was not [48]. Qing et al. showed that, in response to IFN-gamma, SCR-family kinases had to activate STAT3 (but not STAT1) via tyrosine phosphorylation [19]. Some mechanisms that contribute to the specific signal responses of IFN-gamma and IL-6 were not considered in this study. Second, our computational model was a simplification of a biological process and it might not reflect the true regulatory mechanism for the signal interactions. Third, the simulation results in this study were affected by assumptions made based on our experience, e.g., the STAT1/3 heterodimers could be dephosphorylated by PP1 and PP2 in the cytoplasm and nucleus, respectively. Fourth, our model had very rich dynamics parameters that were not fully explored in this study. For simplicity, protein turnover, receptor recycling, de novo synthesis and the degradation of transcription factors were not included in the current model. The ultimate aim of our research is to construct a universal model that accurately reflects the crosstalk between IFN-gamma and IL-6 signals. However, the signal responses to cytokines may be cell type-dependent. Caldenhoven et al. reported that the activation of STAT3 induced by IFN-gamma was lineage-specific in human neutrophils [58]. Zhang et al. showed that IL-6 stimulation could induce STAT1 phosphorylation in a dose- and time-dependent manner in M1, R2 and U937 cells, although it had little effect on STAT1 phosphorylation in 7TD1 and TF1 cells [21]. Bluyssen et al. argued that IL-6 did not activate STAT1 in EC [18]. Furthermore, the concentrations of molecules within the JAK/STAT pathway, such as STATs, are cell type-dependent [6]. The experimental data are limited and not systematic, so we had to create our model based on experimental observations of many different cell types, while we neglected certain contradictory experimental observations. Therefore, it is not possible to expect that the dynamics predicted by our model will apply universally to all types of cells. Thus, structure and parameters of the model may need some adjustment to reflect signal transduction by IFN-gamma and IL-6 in certain cell types. Finally, our crosstalk model was based on experimentally established interactions, but further experimental verification and improvements are required.

Our simulation results showed that STAT1/3 heterodimers have three important functions during signal transductions from IFN-gamma to IL-6. First, the formation of STAT1/3 heterodimers enhances the preferential signal transduction by IFN-gamma and IL-6 because it sequesters a fraction of STAT1* and STAT3*. Second, the formation of STAT1/3 heterodimers prevents mutual reinforcement between IFN-gamma and IL-6 signalling. Finally, the formation of STAT1/3 heterodimers limits the reciprocal inhibition of IFN-gamma and IL-6 signalling. In our simulations, therefore, the formation of STAT1/3 heterodimers dramatically affected the interaction between the IFN-gamma and IL-6 systems, which suggests that STAT1/3 heterodimers may be a potential target for rectifying abnormal signal transduction by IFN-gamma and IL-6. The functional interference of STAT3 homodimers using STAT3 transcription decoys or small molecules in structure-activity relationship (SAR) studies could successfully inhibit the growth of tumour cells [59,60]. However, the therapeutic potential of altering the formation of STAT1/3 heterodimers has not been fully investigated. Thus, further research is still required.

This is the first effort to construct a mathematical model of the crosstalk between IFN and IL-6 signalling. Moreover, our simulation results and theoretical findings provide new insights into the dynamical integration of IFN and IL-6 signals. The lack of experimental data and our current superficial understanding of signal transduction mean there are still many defects in our crosstalk model. We will follow current studies related to this issue and improve the quality and veracity of model in our future research.

## Conclusions

Based on previous models and new experimental observations, we developed the first crosstalk model of IFN-gamma and IL-6 signalling. This theoretical study successfully reproduced key experimental findings and reached some definitive conclusions. First, the unbalanced competition between STAT1 and STAT3 for IFNR and gp130 led to preferential activation of IFN-gamma and IL-6. At the same time, the formation of STAT1/3 heterodimers enhanced preferential signal transduction by sequestering a fraction of STAT1* and STAT3*. Moreover, SOCSs with SHP-2 limited the concentration of the activated receptor complexes of IFN-gamma and IL-6, which also contributed to the preferential activation of IFN-gamma and IL-6. Second, the unbalanced competition between STAT3 and STAT1 was the pivotal mechanism during the mutual switch between IFN-gamma and IL-6 signals after knocking out STAT1 or STAT3. Finally, the formation of STAT1/3 heterodimers prevented the mutual reinforcement of IFN-gamma and IL-6 signalling, and also limited the reciprocal inhibition between IFN-gamma and IL-6 signalling. Moreover, the process of STAT1 homodimer induction of SOCS3 also contributed to asymmetric interactions between IFN-gamma and IL-6 signalling.

## Methods

Three classes of kinetics were involved in our crosstalk model. The mass-action equation for the molecular combination and decomposition was as follows:

$$A+B\overset{K_{f}}{\underset{K_{r}}{⇄}}C+D$$

where *K*_f_ and *K*_r_ are the rates of the forward and reverse reactions. The Michaelis-Menten function (actually be split into two Mass-Action reactions) for the enzymatic reactions was as follows:

$$E+S\overset{K_{1}}{\underset{K_{2}}{⇄}}\mathrm{ES}\overset{K_{3}}{\to }E+P$$

where *E* is the enzyme; *S* is the substrate; *ES* is the enzyme-substrate complex; *P* is the product; and *K*_1_, *K*_2_ and *K*_3_ are the rates of the reactions. The equation for substances undergoing translocation was as follows:

$$A_{C}\overset{K_{f}B}{\to }A_{N}$$

where *A*_*C*_ represents a molecule in the cytoplasm; *A*_*N*_ is *A*_*C*_ translocated from the cytoplasm to nuclei; *B* is the catalyst; and *K*_*f*_ is the rate of the reaction.

**Figure 9:**
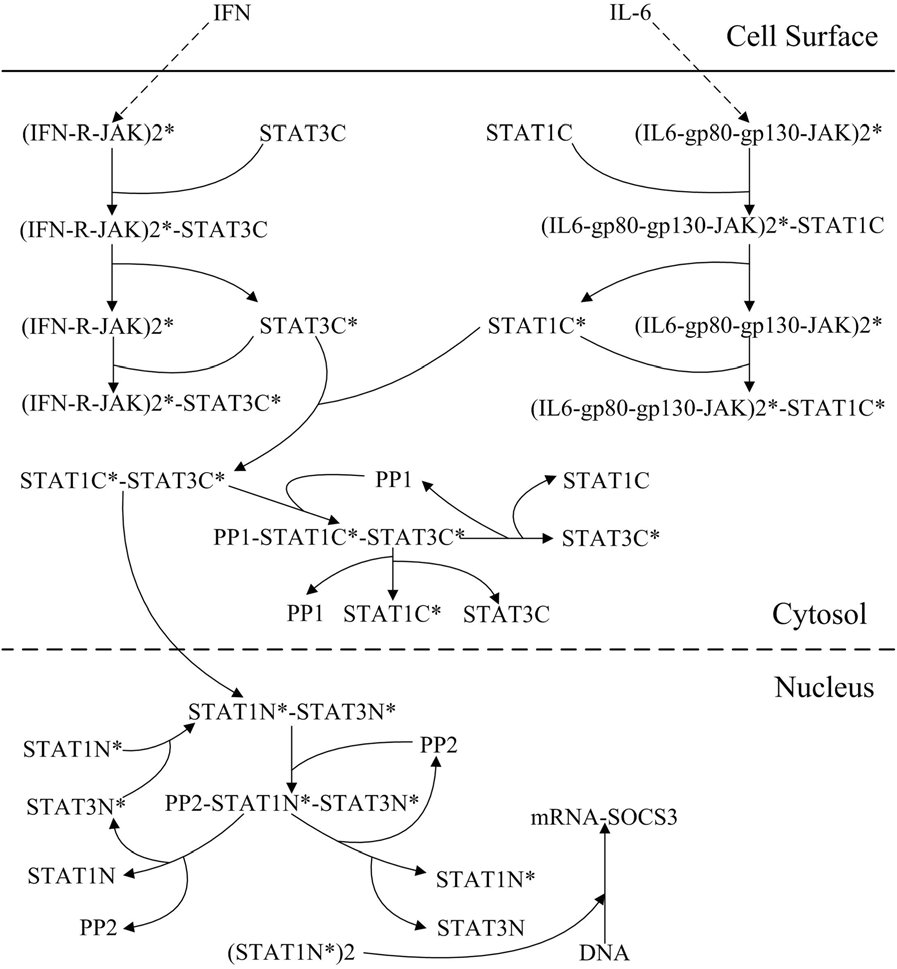
**Schematic description of the newly added biochemical reactions in the crosstalk model.** In the diagram: R represents a receptor; C represents a species in the cytoplasm; N represents a species in the nucleus; 2 represents dimers; and * represents the activation state. mRNA-SOCS3N represents the SOCS3 mRNA in the nucleus. Arrows indicate the reaction direction.

Based on Yamada et al. [28] and Moya et al. [31], we established the components of the IFN-gamma and IL-6 pathways, respectively, where the model parameters of the two components were left unchanged. The equations of these reactions are shown in Additional file 1 and the kinetic parameters of these reactions are provided in Additional file 1: Table S2-S3. Based on the possible physiological mechanisms of IFN-gamma and IL-6 cross-regulation, we also added 16 new reactions (N1)-(N16) in our crosstalk model. The equations of these reactions are listed below (New biochemical reactions added to the crosstalk model) while a graphic description of these reaction is shown in Figure 9. The kinetic parameters of these new reactions are provided in Additional file 1: Table S1.

### New biochemical reactions added to the crosstalk model

STAT3C binding: this positive reaction describes STAT3C binding with the receptor complex of IFN-gamma while the reverse reaction describes the dissociation of STAT3C.

(N1) $$\begin{array}{l} \left(\mathrm{IFN}-R-\mathrm{JAK}\right)2\\ \;*+\mathrm{STAT}3C\overset{n\_\mathrm{kf}1}{\underset{n\_\mathrm{kr}1}{⇄}}\left(\mathrm{IFN}-R-\mathrm{JAK}\right)2\\ \;*-\mathrm{STAT}3C \end{array}$$

STAT3C activation: this reaction describes the activation of STAT3C by the receptor complex of IFN-gamma.

(N2) $$\begin{array}{l} \left(\mathrm{IFN}-R-\mathrm{JAK}\right)2\\ \;*-\mathrm{STAT}3C\overset{n\_k2}{\to }\left(\mathrm{IFN}-R-\mathrm{JAK}\right)2\\ \;*+\mathrm{STAT}3C* \end{array}$$

STAT3C* binding: this positive reaction describes STAT3C* binding with the receptor complex of IFN-gamma while the reverse reaction describes the dissociation of STAT3C*.

(N3) $$\begin{array}{l} \left(\mathrm{IFN}-R-\mathrm{JAK}\right)2*+\mathrm{STAT}3C\\ \;*\overset{n\_\mathrm{kf}3}{\underset{n\_\mathrm{kr}3}{⇄}}\left(\mathrm{IFN}-R-\mathrm{JAK}\right)2*-\mathrm{STAT}3C* \end{array}$$

STAT1C binding: this positive reaction describes STAT1C binding with the receptor complex of IL-6 while the reverse reaction describes the dissociation of STAT1C.

(N4) $$\begin{array}{l} \left(\mathrm{IL}6-\mathrm{gp}80-\mathrm{gp}130-\mathrm{JAK}\right)2\\ \;*+\mathrm{STAT}1C\overset{n\_\mathrm{kf}4}{\underset{n\_\mathrm{kr}4}{⇄}}\left(\mathrm{IL}6-\mathrm{gp}80-\mathrm{gp}130-\mathrm{JAK}\right)2\\ \;*-\mathrm{STAT}1C \end{array}$$

STAT1C activation: this reaction describes the activation of STAT3C by the receptor complex of IL-6.

(N5) $$\begin{array}{l} \left(\mathrm{IL}6-\mathrm{gp}80-\mathrm{gp}130-\mathrm{JAK}\right)2\\ \;*-\mathrm{STAT}1C\overset{n\_k5}{\to }\left(\mathrm{IL}6-\mathrm{gp}80-\mathrm{gp}130-\mathrm{JAK}\right)2\\ \;*+\mathrm{STAT}1C* \end{array}$$

STAT1C* binding: this positive reaction describes STAT3C* binding with the receptor complex of IL-6 while the reverse reaction describes the dissociation of STAT1C*.

(N6) $$\begin{array}{l} \left(\mathrm{IL}6-\mathrm{gp}80-\mathrm{gp}130-\mathrm{JAK}\right)2*+\mathrm{STAT}1C\\ \;*\overset{n\_\mathrm{kf}6}{\underset{n\_\mathrm{kr}6}{⇄}}\left(\mathrm{IL}6-\mathrm{gp}80-\mathrm{gp}130-\mathrm{JAK}\right)2\\ \;*-\mathrm{STAT}1C* \end{array}$$

mRNA-SOCS3 transcript: this reaction describes SOCS3 mRNA transcription after its induction by STAT1 homodimers in the nucleus.

(N7) $$\begin{array}{l} d\left[\mathrm{mRNA}-\mathrm{SOCS}3N\right]/\mathrm{dt}\\ \;=V\max\left(\mathrm{STAT}1N*\right)2/\left[\left(\mathrm{STAT}1N*\right)2+\mathrm{Km}\right] \end{array}$$

STAT1/3 heterodimer formation: this positive reaction describes the formation of STAT1/3 heterodimers in the cytoplasm while the reverse reaction describes their dissociation.

(N8) $$\begin{array}{l} \mathrm{STAT}1C*+\mathrm{STAT}3C*\overset{n\_\mathrm{kf}8}{\underset{n\_\mathrm{kr}8}{⇄}}\mathrm{STAT}1C\\ \;*-\mathrm{STAT}3C* \end{array}$$

STAT1/3 heterodimer formation: this positive reaction describes the formation of STAT1/3 heterodimers in the nucleus while the reverse reaction describes their dissociation.

(N9) $$\begin{array}{l} \mathrm{STAT}1N*+\mathrm{STAT}3N*\overset{n\_\mathrm{kf}9}{\underset{n\_\mathrm{kr}9}{⇄}}\mathrm{STAT}1N\\ \;*-\mathrm{STAT}3N* \end{array}$$

STAT1/3 heterodimer translocation: this reaction describes the nuclear importation of STAT1/3 heterodimers.

(N10) $$\begin{array}{l} \mathrm{STAT}1C*-\mathrm{STAT}3C*\overset{n\_k10}{\to }\mathrm{STAT}1N\\ \;*-\mathrm{STAT}3N* \end{array}$$

PP1 binding: this positive reaction describes PP1 binding with STAT1/3 heterodimers in the cytoplasm while the reverse reaction describes their dissociation.

(N11) $$\begin{array}{l} \mathrm{PP}1+\mathrm{STAT}1C*-\mathrm{STAT}3C*\overset{n\_\mathrm{kf}11}{\underset{n\_\mathrm{kr}11}{⇄}}\mathrm{PP}1\\ \;-\mathrm{STAT}1C*-\mathrm{STAT}3C* \end{array}$$

PP2 binding: this positive reaction describes PP2 binding with STAT1/3 heterodimers in the nucleus while the reverse reaction describes their dissociation.

(N12) $$\begin{array}{l} \mathrm{PP}2+\mathrm{STAT}1N*-\mathrm{STAT}3N*\overset{n\_\mathrm{kf}12}{\underset{n\_\mathrm{kr}12}{⇄}}\mathrm{PP}2\\ \;-\mathrm{STAT}1N*-\mathrm{STAT}3N* \end{array}$$

STAT1C inactivation: this reaction describes the inactivation of STAT1 in STAT1/3 heterodimers by PP1 in the cytoplasm.

(N13) $$\begin{array}{l} \mathrm{PP}1-\mathrm{STAT}1C*-\mathrm{STAT}3C*\overset{n\_k13}{\to }\mathrm{PP}1\\ \;+\mathrm{STAT}1C+\mathrm{STAT}3C* \end{array}$$

STAT3C inactivation: this reaction describes the inactivation of STAT3 in STAT1/3 heterodimers by PP1 in the cytoplasm.

(N14) $$\begin{array}{l} \mathrm{PP}1-\mathrm{STAT}1C*-\mathrm{STAT}3C*\overset{n\_k14}{\to }\mathrm{PP}1\\ \;+\mathrm{STAT}1C*+\mathrm{STAT}3C \end{array}$$

STAT1N inactivation: this reaction describes the inactivation of STAT1 in STAT1/3 heterodimers by PP2 in the nucleus.

(N15) $$\begin{array}{l} \mathrm{PP}2-\mathrm{STAT}1N*-\mathrm{STAT}3N*\overset{n\_k15}{\to }\mathrm{PP}2\\ \;+\mathrm{STAT}1N+\mathrm{STAT}3N* \end{array}$$

STAT3N inactivation: this reaction describes the inactivation of STAT3 in STAT1/3 heterodimers by PP2 in the nucleus.

(N16) $$\begin{array}{l} \mathrm{PP}2-\mathrm{STAT}1N*-\mathrm{STAT}3N*\overset{n\_k16}{\to }\mathrm{PP}2\\ \;+\mathrm{STAT}1N*+\mathrm{STAT}3N \end{array}$$

where R represents the receptor; C represents a species within the cytoplasm; N represents a species within nuclei; 2 represents dimers; and * represents the activation state. mRNA-SOCS3N represents the SOCS3 mRNA in the nucleus; (STAT1N*)2 represents phosphorylated STAT1 homodimers in the nucleus; *V*_max_ represents the maximum reaction velocity of the SOCS3-mRNA transcription; *K*_m_ represents half of the substrate concentration when the enzymatic reaction reaches its maximum reaction velocity.

Using this reaction scenario, we established systematic models that were described using ordinary differential equations (ODEs). COPASI (http://www.copasi.org/) and CellDesigner (http://www.celldesigner.org/) were used to calculate the model states and solve the ODE models [61,62]. Detailed descriptions of the model written in COPSI (using the filename extension “.cps”) are available in Additional file 2.

### Sensitivity analysis

The sensitivity analysis determined the change in the output after a tiny perturbation in the initial state or the reaction parameters. Thus, a perturbation Δ*x* in the parameter set induced an overall state change Δ*y* and the sensitivity was defined as Δ*y*/Δ*x*, which was normalized with respect to the actual values of the phenomenon investigated and the parameter *x/y*. Experimental studies have shown that phosphorylated STAT dimers in the nucleus are critical transcription factors for target gene activation in the JAK/STAT pathway. Therefore, our sensitivity analysis considered (STAT1N*)2 and (STAT3N*)2 as the outputs of IFN-gamma and IL-6 signalling, respectively. In this study, Δ*y* was defined as the change in the concentration of (STAT1N*)2 or (STAT3N*)2 after 1.5 h, which adequately represented the signal transduction strength of IFN-gamma or IL-6. The perturbation ranges of each parameter were set as 30% of the actual absolute value to avoid errors in the sensitivity estimates. COPASI and CellDesigner were used to calculate the sensitivity.

## Competing interests

The authors declare that they have no competing financial interests

## Authors’ contributions

YXH, and YFQ conceived and designed the research. HYW, YZ, YW, CLY, ZBS, LHZ, YS and GNW performed the research including data collection, test and analysis. YLB and LGS suggested extension and modifications to the research. YXL supervised the whole research and revised the manuscript critically. All authors have read and approved the final manuscript.

## Supplementary Material

Additional file 1 **This file contains one figure, nine tables, and description of the biochemical reactions in the crosstalk model and the non-competitive model. Figure S1** shows the overall structure of the crosstalk model. **Table S1** lists the parameters and values of the newly added reactions in the crosstalk model. **Table S2** lists the parameters and values of the IFN-gamma part of the crosstalk model. **Table S3** lists the parameters and values of the IL-6 part of the crosstalk model. **Table S4** lists the state variables of the model and their initial values. **Table S5** lists the re-estimated parameters and their values in the non-competitive model. **Table S6** lists the results of the sensitivity analysis for variations in the concentrations of the pathway components in the competition model. **Table S7** lists the results of the sensitivity analysis for variations in the kinetic parameters in the competition model. **Table S8** lists the results of the sensitivity analysis for variations in the concentrations of pathway components in the non-competitive model. **Table S9** lists the results of the sensitivity analysis for variations in the kinetic parameters in the non-competitive model. All of the biochemical reactions investigated in this study are described in this file, which contains the newly added biochemical reactions, the IFN-gamma and IL-6 parts of the crosstalk model, and the newly added reactions in the non-competitive model. Click here for file

Additional file 2 This file contains the crosstalk model and the non-competitive model written in COPASI, which can be executed using COPASI version 4.7. Click here for file
