# Supplementary material for: Elucidating the crosstalk mechanism between IFN-gamma and IL-6 via mathematical modelling
Source: BMC Bioinformatics. 2013 Feb 6;14:41. doi: 10.1186/1471-2105-14-41 (PMC3599299; doi:10.1186/1471-2105-14-41)
Supplement: Additional file 1 — This file contains one figure, nine tables, and description of the biochemical reactions in the crosstalk model and the non-competitive model. Figure S1 shows the overall structure of the crosstalk model. Table S1 lists the parameters and values of the newly added reactions in the crosstalk model. Table S2 lists the parameters and values of the IFN-gamma part of the crosstalk model. Table S3 lists the parameters and values of the IL-6 part of the crosstalk model. Table S4 lists the state variables of the model and their initial values. Table S5 lists the re-estimated parameters and their values in the non-competitive model. Table S6 lists the results of the sensitivity analysis for variations in the concentrations of the pathway components in the competition model. Table S7 lists the results of the sensitivity analysis for variations in the kinetic parameters in the competition model. Table S8 lists the results of the sensitivity analysis for variations in the concentrations of pathway components in the non-competitive model. Table S9 lists the results of the sensitivity analysis for variations in the kinetic parameters in the non-competitive model. All of the biochemical reactions investigated in this study are described in this file, which contains the newly added biochemical reactions, the IFN-gamma and IL-6 parts of the crosstalk model, and the newly added reactions in the non-competitive model. [file 1471-2105-14-41-S1.pdf]

Figure S1 – The Flow Chart of the Crosstalk Model of IFN-gamma and IL-6 Signalling

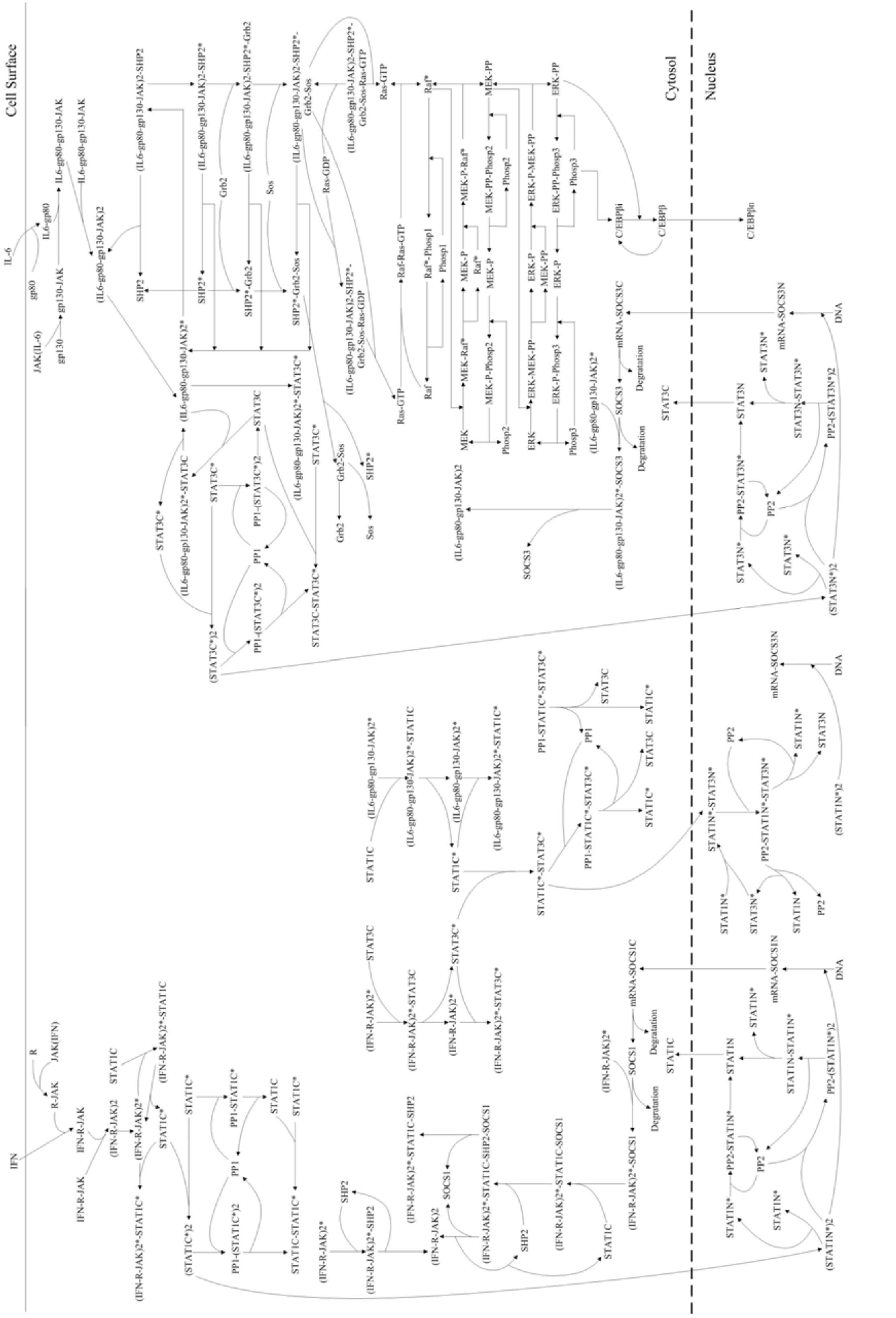

Table S1. Parameters of new adding reactions

Parameter	Value	Description	Reference
n_kf1	0.001	STAT3 binding at IFNR sites	Estimated
n_kr1	7.999422	STAT3 dissociation from IFNR sites	Estimated
n_k2	3.999995	STAT3 activation rate at IFNR sites	Estimated
n_kf3	0.00051	STAT3C* binding at IFNR sites	Estimated
n_kr3	4.982769	STAT3C* dissociation from IFNR sites	Estimated
n_kf4	0.002	STAT1 binding at gp130 sites	Estimated
n_kr4	7.994572	STAT1 dissociation from gp130 sites	Estimated
n_k5	3.999987	STAT1 activation rate at gp130 sites	Estimated
n_kf6	0.000501	STAT1C* binding at gp130 sites	Estimated
n_kr6	4.992048	STAT1C* dissociation from gp130 sites	Estimated
n_kf7	0.001	Max rate of (STAT1N*)2 inducing mRNA-SOCS3	Estimated
n_kr7	400	Km of (STAT1N*)2 inducing mRNA -SOCS3	[1]
n_kf8	0.02	STAT1/3 dimers formation in cytosol	[1-2]
n_kr8	0.1	STAT1/3 dimers dissociation in cytosol	[1-2]
n_kf9	0.02	STAT1/3 dimers formation in nucleus	[2]
n_kr9	0.1	STAT1/3 dimers dissociation in nucleus	[2]
n_k10	0.005	STAT1/3 dimers nuclear import	[1-2]
n_kf11	0.001	PP1 combination with STAT1/3 dimers	[1-2]
n_kr11	0.2	PP1 dissociation from STAT1/3 dimers	[1-2]
n_kf12	0.001	PP2 combination with STAT1/3 dimers	[1-2]
n_kr12	0.2	PP2 dissociation from STAT1/3 dimers	[1-2]
n_k13	0.0015	STAT1 inactivation by PP1 within STAT1/3 dimers	Estimated
n_k14	0.0015	STAT3 inactivation by PP1 within STAT1/3 dimers	Estimated
n_k15	0.0025	STAT1 inactivation by PP2 within STAT1/3 dimers	Estimated
n_k16	0.0025	STAT3 inactivation by PP2 within STAT1/3 dimers	Estimated

First order rate constants are in units of s^{-1} and second order rate constants are expressed in $nM^{-1}s^{-1}$. Michaelis constants are in nM.

TableS2. Parameters of the IFNγ signalling part of the crosstalk model

Parameter	Value	Description	Reference
kf1	0.1	IFNR combination with JAK	[1]

kb1	0.05	IFNR dissociation from JAK	[1]
kf2	0.02	IFN combination with R-JAK	[1]
kb2	0.02	IFN dissociation from R-JAK	[1]
kf3	0.04	(IFN-R-JAK)2 complex dimerization	[1]
kb3	0.2	(IFN-R-JAK)2 complex dissociation	[1]
kf4	0.005	(IFN-R-JAK)2 complex activation	[1]
kf5	0.008	STAT1 binding at IFNR sites	[1]
kb5	0.8	STAT1 dissociation from IFNR sites	[1]
kf6	0.4	STAT1 activation rate at IFNR sites	[1]
kf7	0.005	STAT1C* binding at IFNR sites	[1]
kb7	0.5	STAT1C* dissociation from IFNR sites	[1]
kf8	0.02	STAT1 homodimers formation in cytosol	[1]
kb8	0.1	STAT1 homodimers dissociation in cytosol	[1]
kf9	0.001	SHP2 binding at (IFN-R-JAK)2* complex	[1]
kb9	0.2	SHP2 dissociation from (IFN-R-JAK)2* complex	[1]
kf10	0.003	(IFN-R-JAK)2* complex inactivation of by SHP2	[1]
kf11	0.001	PP1 combination with STAT1C*	[1]
kb11	0.2	PP1 dissociation from STAT1C*	[1]
kf12	0.003	STAT1 inactivation by PP1	[1]
kf13	2.00E-07	STAT1C combination with STAT1C*	[1]
kb13	0.2	STAT1C dissociation from STAT1C*	[1]
kf14	0.005	STAT1 homodimers nuclear import	[1]
kf15	0.001	PP2 combination with STAT1N*	[1]
kb15	0.2	PP2 dissociation from STAT1N*	[1]
kf16	0.005	STAT1 inactivation by PP2	[1]
kf17	0.05	STAT1 nuclear export	[1]
k18a	0.01	Max rate of (STAT1N*)2 inducing mRNA-SOCS1	[1]
k18b	400	Km of (STAT1N*)2 inducing mRNA -SOCS1	[1]
kf19	0.001	mRNA of SOCS1 nuclear export	[1]
kf20	0.01	Translation of SOCS1	[1]
kf21	0.02	SOCS1 binding at (IFN-R-JAK)2* complex	[1]
kb21	0.1	SOCS1 dissociation from (IFN-R-JAK)2* complex	[1]
kf22	0.0005	mRNA of SOCS1 degrading rate	[1]
kf23	0.0005	SOCS1 degrading rate	[1]
kf24	0.001	PP1 combination with (STAT1C*)2	[1]
kb24	0.2	PP1 dissociation from (STAT1C*)2	[1]
kf25	0.003	STAT1 inactivation by PP1 within (STAT1C*)2	[1]
kf26	0.005	STAT1 homodimers formation in nuclear	[1]

kb26	0.5	STAT1 homodimers dissociation in nuclear	[1]
kf27	0.001	PP2 combination with (STAT1N*)2	[1]
kb27	0.2	PP2 dissociation from (STAT1N*)2	[1]
kf28	0.005	STAT1 deactivation by PP2 within (STAT1N*)2	[1]
kf29	2.00E-07	STAT1N combination with STAT1N*	[1]
kb29	0.2	STAT1N dissociation from STAT1N*	[1]
kf30	0.008	STAT1C binding at (IFN-R-JAK)2*-SOCS1 complex	[1]
kb30	0.8	STAT1C dissociation from (IFN-R-JAK)2*-SOCS1 complex	[1]
kf31	0.001	SHP2 binding at (IFN-R-JAK)2*-STAT1C-SOCS1 complex	[1]
kb31	0.2	SHP2 dissociation from (IFN-R-JAK)2*-STAT1C-SOCS1 complex	[1]
kf32	0.003	(IFN-R-JAK)2*-STAT1C-SHP2-SOCS1 complex dissociation	[1]
kf33	0.0005	SOCS1 dissociation from (IFN-R-JAK)2*-STAT1C-SHP2-SOCS1 complex	[1]

First order rate constants are in units of s^{-1} and second order rate constants are expressed in $nM^{-1}s^{-1}$. Michaelis constants are in nM.

Table S3. Parameters of the IL-6 signalling part of the crosstalk model

Parameter	Value	Description	Reference
k1f	0.1	IL6 combination with gp80	[2]
k1r	0.05	IL6 dissociation from gp80	[2]
k2f	0.1	gp130 combination with JAK	[2]
k2r	0.05	gp130 dissociation from JAK	[2]
k3f	0.02	IL6-gp80-gp130-JAK formation	[2]
k3r	0.02	IL6-gp80-gp130-JAK dissociation	[2]
k6f	0.04	(IL6-gp80-gp130-JAK)2 complex dimerization	[2]
k6r	0.2	(IL6-gp80-gp130-JAK)2 complex dissociation	[2]
k7	0.005	(IL6-gp80-gp130-JAK)2 complex activation	[2]
k8f	0.008	STAT3 binding at gp130 sites	[2]
k8r	0.8	STAT3 dissociation from gp130 sites	[2]
k9	0.4	STAT3 activation rate at gp130 sites	[2]
k10f	0.005	STAT3C* binding at gp130 sites	[2]
k10r	0.5	STAT3C* dissociation from gp130 sites	[2]
k16f	0.02	STAT3 homodimers formation in cytosol	[2]
k16r	0.1	STAT3 homodimers dissociation in cytosol	[2]

k17f	0.001	SHP2 binding at (IL6-gp80-gp130-JAK)2* complex	[2]
k17r	0.2	SHP2 dissociation from (IL6-gp80-gp130-JAK)2* complex	[2]
k18	0.003	(IL6-gp80-gp130-JAK)2* complex inactivation of by SHP2	[2]
k19f	0.001	PP1 combination with STAT3C*	[2]
k19r	0.2	PP1 dissociation from STAT3C*	[2]
k20	0.003	STAT3 inactivation by PP1	[2]
k21f	0.001	PP1 combination with (STAT3C*)2	[2]
k21r	0.2	PP1 dissociation from (STAT3C*)2	[2]
k22	0.003	STAT3 inactivation by PP1 within (STAT3C*)2	[2]
k23f	2.00E-07	STAT3C combination with STAT3C*	[2]
k23r	0.2	STAT3C dissociation from STAT3C*	[2]
k24	0.005	STAT3 homodimers nuclear import	[2]
k25f	0.1	STAT1 homodimers dissociation in nuclear	[2]
k25r	0.02	STAT1 homodimers formation in nuclear	[2]
k26f	0.001	PP1 combination with STAT3C*	[2]
k26r	0.2	PP1 dissociation from STAT3C*	[2]
k27	0.005	STAT1 inactivation by PP1	[2]
k28f	0.001	PP1 combination with (STAT3C*)2	[2]
k28r	0.2	PP1 dissociation from (STAT3C*)2	[2]
k29	0.005	STAT3 inactivation by PP1 within (STAT3C*)2	[2]
k30f	0.2	STAT3N dissociation from STAT3N*	[2]
k30r	2.00E-07	STAT3N combination with STAT3N*	[2]
k31	0.05	STAT3 nuclear export	[2]
k32f	0.01	Max rate of (STAT3N*)2 inducing mRNA of SOCS3	[2]
k32r	400	Km of (STAT3N*)2 inducing mRNA of SOCS3	[2]
k33	0.001	mRNA of SOCS3 nuclear export	[2]
k34	0.01	Translation of SOCS3	[2]
k35f	5	SOCS3 binding at (IL6-gp80-gp130-JAK)2* complex	[2]
k35r	0.1	SOCS3 dissociation from (IL6-gp80-gp130-JAK)2* complex	[2]
k36	0.0005	mRNA of SOCS3 degrading rate	[2]
k37	0.0006	SOCS3 degrading rate	[2]
k38	0.003	(IL6-gp80-gp130-JAK)2* complex inactivation of by SHP2	[2]
k39f	6	SHP2 activation within	[2]

		(IL6-gp80-gp130-JAK)2*-SHP2 complex	
k39r	0.06	SHP2 deactivation within (IL6-gp80-gp130-JAK)2*-SHP2 complex	[2]
k40f	0.01	Grb2 binding at (IL6-gp80-gp130-JAK)2*-SHP2* complex	[2]
k40r	0.55	Grb2 dissociation from (IL6-gp80-gp130-JAK)2*-SHP2 complex	[2]
k41f	0.01	SOS binding at (IL6-gp80-gp130-JAK)2*-SHP2*-Grb2 complex	[2]
k41r	0.0214	SOS dissociation from (IL6-gp80-gp130-JAK)2*-SHP2-Grb2 complex	[2]
k42f	0.015	Ras-GDP binding at (IL6-gp80-gp130-JAK)2*-SHP2*-Grb2-SOS complex	[2]
k42r	1.3	Ras-GDP dissociation from (IL6-gp80-gp130-JAK)2*-SHP2-Grb2-SOS complex	[2]
k43f	0.5	Ras-GDP activation by (IL6-gp80-gp130-JAK)2*-SHP2*-Grb2-SOS complex	[2]
k43r	0.0001	Ras-GTP deactivation by (IL6-gp80-gp130-JAK)2*-SHP2*-Grb2-SOS complex	[2]
k44f	0.001	Raf combination with Ras-GTP	[2]
k44r	0.0053	Raf dissociation from Ras-GTP	[2]
k45f	1	Raf activation by Ras-GTP	[2]
k45r	0.0007	Raf* deactivation by Ras-GTP	[2]
k46f	0.0079	Ras-GTP* binding at (IL6-gp80-gp130-JAK)2*-SHP2*-Grb2-SOS complex	[2]
k46r	0.4	Ras-GTP* dissociation from (IL6-gp80-gp130-JAK)2*-SHP2*-Grb2-SOS complex	[2]
k47f	0.023	(IL6-gp80-gp130-JAK)2*-SHP2*-Grb2-SOS-Ras-GTP complex dissociation	[2]
k47r	0.00022	(IL6-gp80-gp130-JAK)2*-SHP2*-Grb2-SOS-Ras-GTP complex formation	[2]
k48f	0.47	SHP2*-Grb2-SOS binding at (IL6-gp80-gp130-JAK)2* complex	[2]
k48r	0.000245	SHP2*-Grb2-SOS dissociation from (IL6-gp80-gp130-JAK)2* complex	[2]
k49f	0.3	SHP2* dissociation from Grb2-SOS	[2]

k49r	0.021	SHP2* binding to Grb2-SOS	[2]
k50f	0.0015	Grb2-SOS complex dissociation	[2]
k50r	0.0045	Grb2-SOS complex formation	[2]
k51f	1.7	Max rate of SHP2* deactivation	[2]
k51r	340	Km of SHP2* deactivation	[2]
k52f	0.3	SHP2* dissociation from (IL6-gp80-gp130-JAK)2* complex	[2]
k52r	0.0009	SHP2* binding to (IL6-gp80-gp130-JAK)2* complex	[2]
k53f	0.01	SHP2* binding to Grb2	[2]
k53r	0.55	SHP2* dissociation from Grb2	[2]
k54f	0.3	SHP2*-Grb2 dissociation from (IL6-gp80-gp130-JAK)2* complex	[2]
k54r	0.0009	SHP2*-Grb2 binding to (IL6-gp80-gp130-JAK)2* complex	[2]
k55f	0.03	SOS dissociation from SHP2*-Grb2 complex	[2]
k55r	0.064	SOS binding to SHP2*-Grb2 complex	[2]
k56f	0.03	Grb2-SOS binding to (IL6-gp80-gp130-JAK)2*-SHP2 complex	[2]
k56r	0.0429	Grb2-SOS dissociation from (IL6-gp80-gp130-JAK)2*-SHP2 complex	[2]
k57f	0.0717	Raf* combination with Phosp1	[2]
k57r	0.2	Raf* dissociation from Phosp1	[2]
k58	1	Raf deactivation by Phosp1	[2]
k59f	0.011	MEK combination with Raf*	[2]
k59r	0.001833	MEK dissociation from Raf*	[2]
k60	3.5	MEK activation by Raf*	[2]
k61f	0.011	MEK-P combination with Raf*	[2]
k61r	0.001833	MEK-P dissociation from Raf*	[2]
k62	2.9	MEK-P activation by Raf*	[2]
k63f	0.0143	MEK-PP combination with Phosp2	[2]
k63r	0.8	MEK-PP dissociation from Phosp2	[2]
k64	0.058	MEK-PP deactivation by Phosp2	[2]
k65f	0.00025	MEK-P combination with Phosp2	[2]
k65r	0.5	MEK-P dissociation from Phosp2	[2]
k66	0.058	MEK-P deactivation by Phosp2	[2]
k67f	0.00011	ERK combination with MEK-PP	[2]
k67r	0.033	ERK dissociation from MEK-PP	[2]
k68	16	ERK activation by MEK-PP	[2]
k69f	0.00011	ERK-P combination with MEK-PP	[2]
k69r	0.033	ERK-P dissociation from MEK-PP	[2]
k70	6.7	ERK-P activation by MEK-PP	[2]
k71f	0.014	ERK-PP combination with Phosp3	[2]

k71r	0.6	ERK-PP dissociation from Phosp3	[2]
k72	0.27	ERK-PP deactivation by Phosp3	[2]
k73f	0.005	ERK-P combination with Phosp3	[2]
k73r	0.5	ERK-P dissociation from Phosp3	[2]
k74	0.3	ERK-P deactivation by Phosp3	[2]
k75f	0.2335	Max rate of CEBPi activation by ERK-PP	[2]
k75r	20000	Km of CEBPi activation by ERK-PP	[2]
k76	0.0388	CEBP deactivation	[2]
k77f	0.9854	CEBPn formation	[2]
k77r	0.0009	CEBPn dissociation	[2]

First order rate constants are in units of s^{-1} and second order rate constants are expressed in $nM^{-1}s^{-1}$. Michaelis constants are in nM.

Table S4. State variables of the crosstalk model and their initial values

Species	Initial values (nM）
gp80	8
IL6-gp80	0
gp130	0.8
JAK(IL-6)	12
gp130-JAK	0
IL6-gp80-gp130-JAK	0
(IL6-gp80-gp130-JAK)2	0
(IL6-gp80-gp130-JAK)2*	0
STAT3C	1000
(IL6-gp80-gp130-JAK)2*-STAT3C	0
STAT3C*	0
(IL6-gp80-gp130-JAK)2*-STAT3C*	0
(STAT3C*)2	0
SHP2	100
(IL6-gp80-gp130-JAK)2*-SHP2	0
PP1	50
PP1-STAT3C*	0
PP1-(STAT3C*)2	0
STAT3C-STAT3C*	0
(STAT3N*)2	0
STAT3N*	0
PP2	60
PP2-STAT3N*	0
STAT3N	0
PP2-(STAT3N*)2	0

STAT3N-STAT3N*	0
mRNA-SOCS3N	0
mRNA-SOCS3C	0
SOCS3	0
(IL6-gp80-gp130-JAK)2*-SOCS3	0
(IL6-gp80-gp130-JAK)2*-SPH2*	0
Grb2	85
(IL6-gp80-gp130-JAK)2*-SPH2*-Grb2	0
SOS	34
(IL6-gp80-gp130-JAK)2*-SPH2*-Grb2-SOS	0
Ras-GDP	19000
(IL6-gp80-gp130-JAK)2*-SPH2*-Grb2-SOS-Ras-GDP	0
Ras-GTP	0
Raf	67
Raf-Ras-GTP	0
Raf*	0
Ras-GTP*	0
(IL6-gp80-gp130-JAK)2*-SPH2*-Grb2-SOS-Ras-GTP	0
SHP2-Grb2-SOS	0
Grb2-SOS	0
SHP2*	0
SHP2*-Grb2	0
Phosp1	0
Raf*-Phosp1	0
MEK	41667
MEK-Raf*	0
MEK-P	0
MEK-P-Raf*	0
MEK-PP	0
Phosp2	67
MEK-PP-Phosp2	0
MEK-P-Phosp2	0
ERK	35000
ERK-MEK-PP	0
ERK-P	0
ERK-P-MEK-PP	0
ERK-PP	0
Phosp3	16667
ERK-PP-Phosp3	0
ERK-P-Phosp3	0
CEBPi	40493
CEBP	0

CEBPn	0
IL-6	0.1
IFN	0.1
R: receptor for IFN	12
JAK(IFN)	12
R-JAK	0
IFN-R-JAK	0
(IFN-R-JAK)2	0
(IFN-R-JAK)2*	0
STAT1C	1000
(IFN-R-JAK)2*-STAT1C	0
STAT1C*	0
(IFN-R-JAK)2*-STAT1C*	0
(STAT1C*)2	0
PP1-STAT1C*	0
PP1-(STAT1C*)2	0
STAT1C-STAT1C*	0
(STAT1N*)2	0
STAT1N*	0
PP2-STAT1N*	0
STAT1N	0
PP2-(STAT1N*)2	0
STAT1N-STAT1N*	0
mRNA-SOCS1N	0
mRNA-SOCS1C	0
SOCS1	0
(IFN-R-JAK)2*-SOCS1	0
(IFN-R-JAK)2*-SHP2	0
(IFN-R-JAK)2*-STAT1C-SOCS1	0
(IFN-R-JAK)2*-STAT1C-SHP2-SOCS1	0
(IFN-R-JAK)2*-STAT1C-SHP2	0
(IL6-gp80-gp130-JAK)2*-STAT1C	0
(IL6-gp80-gp130-JAK)2*-STAT1C*	0
(IFN-R-JAK)2*-STAT3C	0
(IFN-R-JAK)2*-STAT3C*	0
STAT1C*-STAT3C*	0
STAT1N*-STAT3N*	0
PP1-STAT1C*-STAT3C*	0
PP2-STAT1N*-STAT3N*	0
STAT1*	0
STAT3*	0

Table S5. Re-estimated parameters of the non-competitive model

Parameter	Value	Description	Reference
n_kf1	0.002	STAT3 binding at IFNR sites	Estimated
n_kr1	2	STAT3 dissociation from IFNR sites	Estimated
n_k2	0.2	STAT3 activation rate at IFNR sites	Estimated
n_kf3	0.005	STAT3C* binding at IFNR sites	Estimated
n_kr3	0.5	STAT3C* dissociation from IFNR sites	Estimated
n_kf4	0.002	STAT1 binding at gp130 sites	Estimated
n_kr4	2	STAT1 dissociation from gp130 sites	Estimated
n_k5	0.2	STAT1 activation rate at gp130 sites	Estimated
n_kf6	0.005	STAT1C* binding at gp130 sites	Estimated
n_kr6	0.5	STAT1C* dissociation from gp130 sites	Estimated
n_kf17	0.008	STAT1 binding at IFNR sites	[1]
n_kr17	0.8	STAT1 dissociation from IFNR sites	[1]
n_kf18	0.002	STAT3 binding at IFNR sites	Estimated
n_kr18	2	STAT3 dissociation from IFNR sites	Estimated
n_kf19	0.002	STAT1 binding at gp130 sites	Estimated
n_kr19	2	STAT1 dissociation from gp130 sites	Estimated
n_kf20	0.008	STAT3 binding at gp130 sites	[2]
n_kr20	0.8	STAT3 dissociation from gp130 sites	[2]
n_k21	0.4	STAT1 activation rate at IFNR sites	[1]
n_k22	0.2	STAT3 activation rate at IFNR sites	Estimated
n_k23	0.2	STAT1 activation rate at gp130 sites	Estimated
n_k24	0.4	STAT3 activation rate at gp130 sites	[2]

First order rate constants are in units of s^{-1} and second order rate constants are expressed in $nM^{-1}s^{-1}$. Michaelis constants are in nM.

Table S6. Results of sensitivity analysis with respect to variations in the concentrations of pathway components for the competition model

Parameter	IFN	IL-6
PP2	-1.10702	-1.538
PP1	-0.05275	-0.07177
SHP2	-0.52562	-0.5025
STAT3C	0.000918	1.18097
STAT1C	0.499321	0.103269
R	0.529619	0
gp80	0	0.121868
gp130	0	0.055231
JAK(IFN)	0.529618	0

JAK(IL-6)	0	1.67086
IL-6	1.08949	0
IFN	0	0.121868

The definition of the parameter sensitivity can refer to the "Methods" section in the main text. The positive (or negative) sensitivity values indicate consistent (or opposite) percentage changes in output to the perturbation in the parameter compared to the nominal solution.

Table S7. Results of sensitivity analysis with respect to variations in the kinetic parameter for the competition model

Parameter	IFN	IL-6	Description
k1f	0	0.121868	IL6 combination with gp80
k1r	0	-0.191126	IL6 dissociation from gp80
k2f	0	0.0552303	gp130 combination with JAK
k2r	0	-0.072901	gp130 dissociation from JAK
k3f	0	0.146778	IL6-gp80-gp130-JAK formation
k3r	0	-0.195713	IL6-gp80-gp130-JAK dissociation
k6f	0	0.15858	(IL6-gp80-gp130-JAK)2 complex dimerization
k6r	1.58E-07	-0.172858	(IL6-gp80-gp130-JAK)2 complex dissociation
k7	1.82E-07	0.24868	(IL6-gp80-gp130-JAK)2 complex activation
k8f	1.36E-07	0.765515	STAT3 binding at gp130 sites
k8r	-4.14E-07	-0.442549	STAT3 dissociation from gp130 sites
k9	-9.52E-10	0.529317	STAT3 activation rate at gp130 sites
k10f	-2.10E-06	-0.0021417	STAT3C* binding at gp130 sites
k10r	1.57E-07	0.00166223	STAT3C* dissociation from gp130 sites
k16f	0.00134494	-0.00961244	STAT3 homodimers formation in cytosol
k16r	-0.00112481	0.00920069	STAT3 homodimers dissociation in cytosol
k17f	-4.16E-07	-0.459035	SHP2 binding at (IL6-gp80-gp130-JAK)2* complex
k17r	2.59E-07	0.0197143	SHP2 dissociation from (IL6-gp80-gp130-JAK)2* complex
k18	2.48E-07	-0.000504302	(IL6-gp80-gp130-JAK)2* complex inactivation of by SHP2
k19f	0.000936284	-0.0392736	PP1 combination with STAT3C*
k19r	-0.000763889	0.0312984	PP1 dissociation from STAT3C*
k20	0.00224987	-0.0523803	STAT3 inactivation by PP1
k21f	-0.00067499	-0.0120521	PP1 combination with (STAT3C*)2
k21r	0.000525229	0.00936552	PP1 dissociation from (STAT3C*)2

k22	0.000900784	-0.0598852	STAT3 inactivation by PP1 within (STAT3C*)2
k23f	-8.69E-06	4.08E-05	STAT3C combination with STAT3C*
k23r	1.55E-05	-0.000246455	STAT3C dissociation from STAT3C*
k24	0.00974621	-0.184408	STAT3 homodimers nuclear import
k25f	-0.00543954	-0.152308	STAT1 homodimers dissociation in nuclear
k25r	0.00705188	0.144074	STAT1 homodimers formation in nuclear
k26f	0.0174087	-0.330483	PP1 combination with STAT3C*
k26r	-0.0136058	0.268419	PP1 dissociation from STAT3C*
k27	0.00810629	-0.431082	STAT1 inactivation by PP1
k28f	0.0099286	-0.826581	PP1 combination with (STAT3C*)2
k28r	-0.00784331	0.936416	PP1 dissociation from (STAT3C*)2
k29	0.00526295	-1.15251	STAT3 inactivation by PP1 within (STAT3C*)2
k30f	3.84E-06	-0.000773945	STAT3N dissociation from STAT3N*
k30r	-5.41E-07	7.60E-08	STAT3N combination with STAT3N*
k31	-1.61E-05	0.00166878	STAT3 nuclear export
k32f	1.58E-07	-0.47458	Max rate of (STAT3N*)2 inducing mRNA of SOCS3
k32r	1.20E-10	0.494908	Km of (STAT3N*)2 inducing mRNA of SOCS3
k33	-1.17E-06	-0.327549	mRNA of SOCS3 nuclear export
k34	-2.87E-07	-0.475239	Translation of SOCS3
k35f	1.55E-07	-0.206762	SOCS3 binding at (IL6-gp80-gp130-JAK)2* complex
k35r	1.56E-07	0.242485	SOCS3 dissociation from (IL6-gp80-gp130-JAK)2* complex
k36	4.65E-12	0.10735	mRNA of SOCS3 degrading rate
k37	2.44E-07	0.0598051	SOCS3 degrading rate
k38	1.56E-07	-0.227934	(IL6-gp80-gp130-JAK)2* complex inactivation of by SHP2
k39f	3.76E-10	-0.014523	SHP2 activation within (IL6-gp80-gp130-JAK)2*-SHP2 complex
k39r	2.44E-07	0.000925971	SHP2 deactivation within (IL6-gp80-gp130-JAK)2*-SHP2 complex

k40f	1.56E-07	0.0909505	Grb2 binding at (IL6-gp80-gp130-JAK)2*-SHP2* complex
k40r	-3.14E-07	-0.0551323	Grb2 dissociation from (IL6-gp80-gp130-JAK)2*-SHP2 complex
k41f	2.44E-07	-0.00252796	SOS binding at (IL6-gp80-gp130-JAK)2*-SHP2*-Grb2 complex
k41r	2.44E-07	0.0177295	SOS dissociation from (IL6-gp80-gp130-JAK)2*-SHP2-Grb2 complex
k42f	2.76E-07	-0.483011	Ras-GDP binding at (IL6-gp80-gp130-JAK)2*-SHP2*-Grb2-SOS complex
k42r	4.04E-10	0.407438	Ras-GDP dissociation from (IL6-gp80-gp130-JAK)2*-SHP2-Grb2-SOS complex
k43f	2.44E-07	0.119607	Ras-GDP activation by (IL6-gp80-gp130-JAK)2*-SHP2*-Grb2-SOS complex
k43r	0	-5.07E-06	Ras-GTP deactivation by (IL6-gp80-gp130-JAK)2*-SHP2*-Grb2-SOS complex
k44f	0	-0.0014107	Raf combination with Ras-GTP
k44r	0	1.15E-05	Raf dissociation from Ras-GTP
k45f	4.64E-10	-0.000132014	Raf activation by Ras-GTP
k45r	0	2.60E-05	Raf* deactivation by Ras-GTP
k46f	0	-0.0760325	Ras-GTP* binding at (IL6-gp80-gp130-JAK)2*-SHP2*-Grb2-SOS complex
k46r	5.25E-10	0.0881827	Ras-GTP* dissociation from (IL6-gp80-gp130-JAK)2*-SHP2*-Grb2-SOS complex
k47f	1.82E-07	0.00638043	(IL6-gp80-gp130-JAK)2*-SHP2*-Grb2-SOS -Ras-GTP complex dissociation
k47r	1.58E-07	-0.0361897	(IL6-gp80-gp130-JAK)2*-SHP2*-Grb2-SOS -Ras-GTP complex formation
k48f	2.62E-07	0.647503	SHP2*-Grb2-SOS binding at (IL6-gp80-gp130-JAK)2* complex
k48r	0	-0.00781241	SHP2*-Grb2-SOS dissociation from (IL6-gp80-gp130-JAK)2* complex
k49f	1.56E-07	-0.0205943	SHP2* dissociation from Grb2-SOS

k49r	4.78E-15	0.0267126	SHP2* binding to Grb2-SOS
k50f	4.32E-07	0.00123088	Grb2-SOS complex dissociation
k50r	4.36E-07	-0.00430478	Grb2-SOS complex formation
k51f	1.56E-07	-0.0175449	Max rate of SHP2* deactivation
k51r	1.56E-07	0.0167897	Km of SHP2* deactivation
k52f	9.08E-11	0.160398	SHP2* dissociation from (IL6-gp80-gp130-JAK)2* complex
k52r	0	-0.0109974	SHP2* binding to (IL6-gp80-gp130-JAK)2* complex
k53f	1.58E-07	-0.00351982	SHP2* binding to Grb2
k53r	1.58E-07	0.00338257	SHP2* dissociation from Grb2
k54f	1.58E-07	0.062672	SHP2*-Grb2 dissociation from (IL6-gp80-gp130-JAK)2* complex
k54r	0	-0.00768864	SHP2*-Grb2 binding to (IL6-gp80-gp130-JAK)2* complex
k55f	0	0.000118197	SOS dissociation from SHP2*-Grb2 complex
k55r	4.64E-10	-0.000569644	SOS binding to SHP2*-Grb2 complex
k56f	1.56E-07	-0.195653	Grb2-SOS binding to (IL6-gp80-gp130-JAK)2*-SHP2 complex
k56r	2.76E-07	0.0222085	Grb2-SOS dissociation from (IL6-gp80-gp130-JAK)2*-SHP2 complex
k57f	0	-8.37E-05	Raf* combination with Phosp1
k57r	0	7.71E-06	Raf* dissociation from Phosp1
k58	0	-5.07E-05	Raf deactivation by Phosp1
k59f	0	-7.07E-05	MEK combination with Raf*
k59r	0	-6.15E-07	MEK dissociation from Raf*
k60	0	-0.00021936	MEK activation by Raf*
k61f	0	4.85E-05	MEK-P combination with Raf*
k61r	0	1.32E-07	MEK-P dissociation from Raf*
k62	0	-0.000388358	MEK-P activation by Raf*
k63f	0	1.03E-06	MEK-PP combination with Phosp2
k63r	0	-1.60E-06	MEK-PP dissociation from Phosp2
k64	0	4.73E-05	MEK-PP deactivation by Phosp2
k65f	0	9.47E-07	MEK-P combination with Phosp2

k65r	0	-1.04E-06	MEK-P dissociation from Phosp2
k66	0	5.21E-06	MEK-P deactivation by Phosp2
k67f	0	-4.82E-07	ERK combination with MEK-PP
k67r	0	3.90E-07	ERK dissociation from MEK-PP
k68	0	4.95E-07	ERK activation by MEK-PP
k69f	0	-4.01E-07	ERK-P combination with MEK-PP
k69r	0	-1.72E-07	ERK-P dissociation from MEK-PP
k70	0	-3.31E-07	ERK-P activation by MEK-PP
k71f	0	4.35E-08	ERK-PP combination with Phosp3
k71r	0	-1.09E-07	ERK-PP dissociation from Phosp3
k72	0	-5.56E-09	ERK-PP deactivation by Phosp3
k73f	0	-5.10E-07	ERK-P combination with Phosp3
k73r	0	-6.49E-07	ERK-P dissociation from Phosp3
k74	0	-4.61E-08	ERK-P deactivation by Phosp3
k75f	0	-1.45E-07	Max rate of CEBPi activation by ERK-PP
k75r	0	1.61E-07	Km of CEBPi activation by ERK-PP
k76	0	3.91E-07	CEBP deactivation
k77f	0	-6.04E-07	CEBPn formation
k77r	0	-6.10E-07	CEBPn dissociation
kf1	0.108106	-5.76E-08	IFNR combination with JAK
kb1	-0.118735	7.64E-08	IFNR dissociation from JAK
kf2	1.08949	0	IFN combination with R-JAK
kb2	-0.860051	0	IFN dissociation from R-JAK
kf3	0.561679	0	(IFN-R-JAK)2 complex dimerization
kb3	-0.485481	2.59E-07	(IFN-R-JAK)2 complex dissociation
kf4	0.579146	-1.81E-07	(IFN-R-JAK)2 complex activation
kf5	0.920281	3.73E-08	STAT1 binding at IFNR sites
kb5	-0.527806	-7.59E-07	STAT1 dissociation from IFNR sites
kf6	0.494715	-1.71E-07	STAT1 activation rate at IFNR sites
kf7	-0.00191204	-2.31E-07	STAT1C* binding at IFNR sites
kb7	0.00150782	-6.54E-07	STAT1C* dissociation from IFNR sites

kf8	-0.0126352	0.002968	STAT1 homodimers formation in cytosol
kb8	0.0126982	-0.00273999	STAT1 homodimers dissociation in cytosol
kf9	-0.200213	5.17E-08	SHP2 binding at (IFN-R-JAK)2* complex
kb9	0.173514	3.03E-07	SHP2 dissociation from (IFN-R-JAK)2* complex
kf10	-0.197613	-4.03E-07	(IFN-R-JAK)2* complex inactivation of by SHP2
kf11	-0.0290778	-0.000975343	PP1 combination with STAT1C*
kb11	0.022967	0.000590942	PP1 dissociation from STAT1C*
kf12	-0.0416347	0.000488961	STAT1 inactivation by PP1
kf13	4.18E-05	-1.10E-05	STAT1C combination with STAT1C*
kb13	-0.000292798	2.71E-05	STAT1C dissociation from STAT1C*
kf14	-0.190609	0.0201524	STAT1 homodimers nuclear import
kf15	-0.382591	0.110933	PP2 combination with STAT1N*
kb15	0.35352	-0.10356	PP2 dissociation from STAT1N*
kf17	0.00121986	9.73E-06	STAT1 inactivation by PP2
kf16	-0.67097	0.0575866	STAT1 nuclear export
k18a	-0.646078	9.01E-08	Max rate of (STAT1N*)2 inducing mRNA-SOCS1
k18b	0.684174	1.04E-07	Km of (STAT1N*)2 inducing mRNA -SOCS1
kf19	-0.373617	-1.42E-07	mRNA of SOCS1 nuclear export
kf20	-0.646079	1.74E-07	Translation of SOCS1
kf21	-0.59167	2.12E-07	SOCS1 binding at (IFN-R-JAK)2* complex
kb21	0.576228	-7.30E-07	SOCS1 dissociation from (IFN-R-JAK)2* complex
kf22	0.199141	-2.63E-07	mRNA of SOCS1 degrading rate
kf23	0.179613	1.48E-07	SOCS1 degrading rate
kf24	-0.0140464	-0.00367923	PP1 combination with (STAT1C*)2
kb24	0.0107448	0.00288126	PP1 dissociation from (STAT1C*)2
kf25	-0.0639015	-0.00108704	STAT1 inactivation by PP1 within (STAT1C*)2
kf26	0.121735	-0.00220328	STAT1 homodimers formation in nuclear
kb26	-0.118656	0.00212399	STAT1 homodimers dissociation in nuclear

kf27	-0.314663	0.00989138	PP2 combination with (STAT1N*)2
kb27	0.297118	-0.0078173	PP2 dissociation from (STAT1N*)2
kf28	-0.537823	0.00530863	STAT1 deactivation by PP2 within (STAT1N*)2
kf29	8.67E-07	-3.84E-07	STAT1N combination with STAT1N*
kb29	-0.00022888	1.85E-05	STAT1N dissociation from STAT1N*
kf30	-0.474515	-6.65E-07	STAT1C binding at (IFN-R-JAK)2*-SOCS1 complex
kb30	0.543759	-8.42E-08	STAT1C dissociation from (IFN-R-JAK)2*-SOCS1 complex
kf31	-0.349744	-4.44E-07	SHP2 binding at (IFN-R-JAK)2*-STAT1C-SOCS1 complex
kb31	0.34494	-3.07E-07	SHP2 dissociation from (IFN-R-JAK)2*-STAT1C-SOCS1 complex
kf32	-0.229267	-8.59E-08	(IFN-R-JAK)2*-STAT1C-SHP2-SOCS1 complex dissociation
kf33	-0.0576678	-4.44E-07	SOCS1 dissociation from (IFN-R-JAK)2*-STAT1C-SHP2-SOCS1 complex
n_kf1	0.00590598	1.85E-07	STAT3 binding at IFNR sites
n_kr1	-0.00416891	-3.92E-07	STAT3 dissociation from IFNR sites
n_k2	0.00402307	-6.10E-07	STAT3 activation rate at IFNR sites
n_kf3	-6.42E-06	-3.55E-07	STAT3C* binding at IFNR sites
n_kr3	6.53E-06	-8.21E-08	STAT3C* dissociation from IFNR sites
n_kf4	2.76E-07	0.100181	STAT1 binding at gp130 sites
n_kr4	2.76E-07	-0.0591942	STAT1 dissociation from gp130 sites
n_k5	2.42E-07	0.0640334	STAT1 activation rate at gp130 sites
n_kf6	-2.42E-07	-9.89E-06	STAT1C* binding at gp130 sites
n_kr6	1.79E-11	7.80E-06	STAT1C* dissociation from gp130 sites
n_kf7	1.46E-07	-0.00103274	Max rate of (STAT1N*)2 inducing mRNA-SOCS3
n_kr7	2.59E-07	0.000793487	Km of (STAT1N*)2 inducing mRNA -SOCS3
n_kf8	0.00461257	0.00504897	STAT1/3 dimers formation in cytosol
n_kr8	-0.0042064	-0.00436441	STAT1/3 dimers dissociation in cytosol
n_kf9	-0.021741	-0.0671042	STAT1/3 dimers formation in nucleus

n_kr9	0.0264869	0.0613915	STAT1/3 dimers dissociation in nucleus
n_k10	0.00187114	-0.00882142	STAT1/3 dimers nuclear import
n_kf11	-0.0052467	-0.0085852	PP1 combination with STAT1/3 dimers
n_kr11	0.00408078	0.00669567	PP1 dissociation from STAT1/3 dimers
n_kf12	0.0244101	-0.103754	PP2 combination with STAT1/3 dimers
n_kr12	-0.0223063	0.101852	PP2 dissociation from STAT1/3 dimers
n_k13	-0.00882717	-0.00158213	STAT1 inactivation by PP1 within STAT1/3 dimers
n_k14	0.00130904	-0.012887	STAT3 inactivation by PP1 within STAT1/3 dimers
n_k15	-0.196276	0.082933	STAT1 inactivation by PP2 within STAT1/3 dimers
n_k16	0.0742326	-0.423821	STAT3 inactivation by PP2 within STAT1/3 dimers

The definition of the parameter sensitivity can refer to the "Methods" section in the main text. The positive (or negative) sensitivity values indicate consistent (or opposite) percentage changes in output to the perturbation in the parameter compared to the nominal solution.

Table S8. Results of sensitivity analysis with respect to variations in the concentrations of pathway components for the non-competitive model

Parameter	IFN	IL-6
PP2	-0.86819	-1.49525
PP1	-0.05413	-0.06777
SHP2	-0.42032	-0.44196
STAT3C	0.307906	1.36415
STAT1C	0.498613	0.398571
R	0.487952	0
gp80	0	0.126754
gp130	0	0.055787
JAK(IFN)	0.487951	0
JAK(IL-6)	0	1.46179
IL-6	0.964033	0
IFN	0	0.126754

The definition of the parameter sensitivity can refer to the "Methods" section in the main text. The positive (or negative) sensitivity values indicate consistent (or opposite) percentage changes in output to the perturbation in the parameter compared to the nominal solution.

Table S9. Results of sensitivity analysis with respect to variations in the kinetic parameter for the non-competitive model

Parameter	IFN	IL-6	Description
k1f	0	0.121868	IL6 combination with gp80
k1r	0	-0.191126	IL6 dissociation from gp80
k2f	0	0.0552303	gp130 combination with JAK
k2r	0	-0.072901	gp130 dissociation from JAK
k3f	0	0.146778	IL6-gp80-gp130-JAK formation
k3r	0	-0.195713	IL6-gp80-gp130-JAK dissociation
k6f	0	0.15858	(IL6-gp80-gp130-JAK)2 complex dimerization
k6r	1.58E-07	-0.172858	(IL6-gp80-gp130-JAK)2 complex dissociation
k7	1.82E-07	0.24868	(IL6-gp80-gp130-JAK)2 complex activation
k8f	1.36E-07	0.765515	STAT3 binding at gp130 sites
k8r	-4.14E-07	-0.442549	STAT3 dissociation from gp130 sites
k9	-9.52E-10	0.529317	STAT3 activation rate at gp130 sites
k10f	-2.10E-06	-0.0021417	STAT3C* binding at gp130 sites
k10r	1.57E-07	0.00166223	STAT3C* dissociation from gp130 sites
k16f	0.00134494	-0.00961244	STAT3 homodimers formation in cytosol
k16r	-0.00112481	0.00920069	STAT3 homodimers dissociation in cytosol
k17f	-4.16E-07	-0.459035	SHP2 binding at (IL6-gp80-gp130-JAK)2* complex
k17r	2.59E-07	0.0197143	SHP2 dissociation from (IL6-gp80-gp130-JAK)2* complex
k18	2.48E-07	-0.000504302	(IL6-gp80-gp130-JAK)2* complex inactivation of by SHP2
k19f	0.000936284	-0.0392736	PP1 combination with STAT3C*
k19r	-0.000763889	0.0312984	PP1 dissociation from STAT3C*
k20	0.00224987	-0.0523803	STAT3 inactivation by PP1
k21f	-0.00067499	-0.0120521	PP1 combination with (STAT3C*)2
k21r	0.000525229	0.00936552	PP1 dissociation from (STAT3C*)2
k22	0.000900784	-0.0598852	STAT3 inactivation by PP1 within (STAT3C*)2
k23f	-8.69E-06	4.08E-05	STAT3C combination with STAT3C*
k23r	1.55E-05	-0.000246455	STAT3C dissociation from STAT3C*
k24	0.00974621	-0.184408	STAT3 homodimers nuclear import
k25f	-0.00543954	-0.152308	STAT1 homodimers dissociation in nuclear
k25r	0.00705188	0.144074	STAT1 homodimers formation in nuclear

k26f	0.0174087	-0.330483	PP1 combination with STAT3C*
k26r	-0.0136058	0.268419	PP1 dissociation from STAT3C*
k27	0.00810629	-0.431082	STAT1 inactivation by PP1
k28f	0.0099286	-0.826581	PP1 combination with (STAT3C*)2
k28r	-0.00784331	0.936416	PP1 dissociation from (STAT3C*)2
k29	0.00526295	-1.15251	STAT3 inactivation by PP1 within (STAT3C*)2
k30f	3.84E-06	-0.000773945	STAT3N dissociation from STAT3N*
k30r	-5.41E-07	7.60E-08	STAT3N combination with STAT3N*
k31	-1.61E-05	0.00166878	STAT3 nuclear export
k32f	1.58E-07	-0.47458	Max rate of (STAT3N*)2 inducing mRNA of SOCS3
k32r	1.20E-10	0.494908	Km of (STAT3N*)2 inducing mRNA of SOCS3
k33	-1.17E-06	-0.327549	mRNA of SOCS3 nuclear export
k34	-2.87E-07	-0.475239	Translation of SOCS3
k35f	1.55E-07	-0.206762	SOCS3 binding at (IL6-gp80-gp130-JAK)2* complex
k35r	1.56E-07	0.242485	SOCS3 dissociation from (IL6-gp80-gp130-JAK)2* complex
k36	4.65E-12	0.10735	mRNA of SOCS3 degrading rate
k37	2.44E-07	0.0598051	SOCS3 degrading rate
k38	1.56E-07	-0.227934	(IL6-gp80-gp130-JAK)2* complex inactivation of by SHP2
k39f	3.76E-10	-0.014523	SHP2 activation within (IL6-gp80-gp130-JAK)2*-SHP2 complex
k39r	2.44E-07	0.000925971	SHP2 deactivation within (IL6-gp80-gp130-JAK)2*-SHP2 complex
k40f	1.56E-07	0.0909505	Grb2 binding at (IL6-gp80-gp130-JAK)2*-SHP2* complex
k40r	-3.14E-07	-0.0551323	Grb2 dissociation from (IL6-gp80-gp130-JAK)2*-SHP2 complex
k41f	2.44E-07	-0.00252796	SOS binding at (IL6-gp80-gp130-JAK)2*-SHP2*-Grb2 complex
k41r	2.44E-07	0.0177295	SOS dissociation from (IL6-gp80-gp130-JAK)2*-SHP2-Grb2 complex

k42f	2.76E-07	-0.483011	Ras-GDP binding at (IL6-gp80-gp130-JAK)2*-SHP2*-Grb2-SOS complex
k42r	4.04E-10	0.407438	Ras-GDP dissociation from (IL6-gp80-gp130-JAK)2*-SHP2-Grb2-SOS complex
k43f	2.44E-07	0.119607	Ras-GDP activation by (IL6-gp80-gp130-JAK)2*-SHP2*-Grb2-SOS complex
k43r	0	-5.07E-06	Ras-GTP deactivation by (IL6-gp80-gp130-JAK)2*-SHP2*-Grb2-SOS complex
k44f	0	-0.0014107	Raf combination with Ras-GTP
k44r	0	1.15E-05	Raf dissociation from Ras-GTP
k45f	4.64E-10	-0.000132014	Raf activation by Ras-GTP
k45r	0	2.60E-05	Raf* deactivation by Ras-GTP
k46f	0	-0.0760325	Ras-GTP* binding at (IL6-gp80-gp130-JAK)2*-SHP2*-Grb2-SOS complex
k46r	5.25E-10	0.0881827	Ras-GTP* dissociation from (IL6-gp80-gp130-JAK)2*-SHP2*-Grb2-SOS complex
k47f	1.82E-07	0.00638043	(IL6-gp80-gp130-JAK)2*-SHP2*-Grb2-SOS-Ras-GTP complex dissociation
k47r	1.58E-07	-0.0361897	(IL6-gp80-gp130-JAK)2*-SHP2*-Grb2-SOS-Ras-GTP complex formation
k48f	2.62E-07	0.647503	SHP2*-Grb2-SOS binding at (IL6-gp80-gp130-JAK)2* complex
k48r	0	-0.00781241	SHP2*-Grb2-SOS dissociation from (IL6-gp80-gp130-JAK)2* complex
k49f	1.56E-07	-0.0205943	SHP2* dissociation from Grb2-SOS
k49r	4.78E-15	0.0267126	SHP2* binding to Grb2-SOS
k50f	4.32E-07	0.00123088	Grb2-SOS complex dissociation
k50r	4.36E-07	-0.00430478	Grb2-SOS complex formation
k51f	1.56E-07	-0.0175449	Max rate of SHP2* deactivation
k51r	1.56E-07	0.0167897	Km of SHP2* deactivation
k52f	9.08E-11	0.160398	SHP2* dissociation from (IL6-gp80-gp130-JAK)2* complex

k52r	0	-0.0109974	SHP2* binding to (IL6-gp80-gp130-JAK)2* complex
k53f	1.58E-07	-0.00351982	SHP2* binding to Grb2
k53r	1.58E-07	0.00338257	SHP2* dissociation from Grb2
k54f	1.58E-07	0.062672	SHP2*-Grb2 dissociation from (IL6-gp80-gp130-JAK)2* complex
k54r	0	-0.00768864	SHP2*-Grb2 binding to (IL6-gp80-gp130-JAK)2* complex
k55f	0	0.000118197	SOS dissociation from SHP2*-Grb2 complex
k55r	4.64E-10	-0.000569644	SOS binding to SHP2*-Grb2 complex
k56f	1.56E-07	-0.195653	Grb2-SOS binding to (IL6-gp80-gp130-JAK)2*-SHP2 complex
k56r	2.76E-07	0.0222085	Grb2-SOS dissociation from (IL6-gp80-gp130-JAK)2*-SHP2 complex
k57f	0	-8.37E-05	Raf* combination with Phosp1
k57r	0	7.71E-06	Raf* dissociation from Phosp1
k58	0	-5.07E-05	Raf deactivation by Phosp1
k59f	0	-7.07E-05	MEK combination with Raf*
k59r	0	-6.15E-07	MEK dissociation from Raf*
k60	0	-0.00021936	MEK activation by Raf*
k61f	0	4.85E-05	MEK-P combination with Raf*
k61r	0	1.32E-07	MEK-P dissociation from Raf*
k62	0	-0.000388358	MEK-P activation by Raf*
k63f	0	1.03E-06	MEK-PP combination with Phosp2
k63r	0	-1.60E-06	MEK-PP dissociation from Phosp2
k64	0	4.73E-05	MEK-PP deactivation by Phosp2
k65f	0	9.47E-07	MEK-P combination with Phosp2
k65r	0	-1.04E-06	MEK-P dissociation from Phosp2
k66	0	5.21E-06	MEK-P deactivation by Phosp2
k67f	0	-4.82E-07	ERK combination with MEK-PP
k67r	0	3.90E-07	ERK dissociation from MEK-PP
k68	0	4.95E-07	ERK activation by MEK-PP
k69f	0	-4.01E-07	ERK-P combination with MEK-PP

k69r	0	-1.72E-07	ERK-P dissociation from MEK-PP
k70	0	-3.31E-07	ERK-P activation by MEK-PP
k71f	0	4.35E-08	ERK-PP combination with Phosp3
k71r	0	-1.09E-07	ERK-PP dissociation from Phosp3
k72	0	-5.56E-09	ERK-PP deactivation by Phosp3
k73f	0	-5.10E-07	ERK-P combination with Phosp3
k73r	0	-6.49E-07	ERK-P dissociation from Phosp3
k74	0	-4.61E-08	ERK-P deactivation by Phosp3
k75f	0	-1.45E-07	Max rate of CEBPi activation by ERK-PP
k75r	0	1.61E-07	Km of CEBPi activation by ERK-PP
k76	0	3.91E-07	CEBP deactivation
k77f	0	-6.04E-07	CEBPn formation
k77r	0	-6.10E-07	CEBPn dissociation
kf1	0.108106	-5.76E-08	IFNR combination with JAK
kb1	-0.118735	7.64E-08	IFNR dissociation from JAK
kf2	1.08949	0	IFN combination with R-JAK
kb2	-0.860051	0	IFN dissociation from R-JAK
kf3	0.561679	0	(IFN-R-JAK)2 complex dimerization
kb3	-0.485481	2.59E-07	(IFN-R-JAK)2 complex dissociation
kf4	0.579146	-1.81E-07	(IFN-R-JAK)2 complex activation
kf5	0.920281	3.73E-08	STAT1 binding at IFNR sites
kb5	-0.527806	-7.59E-07	STAT1 dissociation from IFNR sites
kf6	0.494715	-1.71E-07	STAT1 activation rate at IFNR sites
kf7	-0.00191204	-2.31E-07	STAT1C* binding at IFNR sites
kb7	0.00150782	-6.54E-07	STAT1C* dissociation from IFNR sites
kf8	-0.0126352	0.002968	STAT1 homodimers formation in cytosol
kb8	0.0126982	-0.00273999	STAT1 homodimers dissociation in cytosol
kf9	-0.200213	5.17E-08	SHP2 binding at (IFN-R-JAK)2* complex
kb9	0.173514	3.03E-07	SHP2 dissociation from (IFN-R-JAK)2* complex
kf10	-0.197613	-4.03E-07	(IFN-R-JAK)2* complex inactivation of by SHP2

kf11	-0.0290778	-0.000975343	PP1 combination with STAT1C*
kb11	0.022967	0.000590942	PP1 dissociation from STAT1C*
kf12	-0.0416347	0.000488961	STAT1 inactivation by PP1
kf13	4.18E-05	-1.10E-05	STAT1C combination with STAT1C*
kb13	-0.000292798	2.71E-05	STAT1C dissociation from STAT1C*
kf14	-0.190609	0.0201524	STAT1 homodimers nuclear import
kf15	-0.382591	0.110933	PP2 combination with STAT1N*
kb15	0.35352	-0.10356	PP2 dissociation from STAT1N*
kf17	0.00121986	9.73E-06	STAT1 inactivation by PP2
kf16	-0.67097	0.0575866	STAT1 nuclear export
k18a	-0.646078	9.01E-08	Max rate of (STAT1N*)2 inducing mRNA-SOCS1
k18b	0.684174	1.04E-07	Km of (STAT1N*)2 inducing mRNA -SOCS1
kf19	-0.373617	-1.42E-07	mRNA of SOCS1 nuclear export
kf20	-0.646079	1.74E-07	Translation of SOCS1
kf21	-0.59167	2.12E-07	SOCS1 binding at (IFN-R-JAK)2* complex
kb21	0.576228	-7.30E-07	SOCS1 dissociation from (IFN-R-JAK)2* complex
kf22	0.199141	-2.63E-07	mRNA of SOCS1 degrading rate
kf23	0.179613	1.48E-07	SOCS1 degrading rate
kf24	-0.0140464	-0.00367923	PP1 combination with (STAT1C*)2
kb24	0.0107448	0.00288126	PP1 dissociation from (STAT1C*)2
kf25	-0.0639015	-0.00108704	STAT1 inactivation by PP1 within (STAT1C*)2
kf26	0.121735	-0.00220328	STAT1 homodimers formation in nuclear
kb26	-0.118656	0.00212399	STAT1 homodimers dissociation in nuclear
kf27	-0.314663	0.00989138	PP2 combination with (STAT1N*)2
kb27	0.297118	-0.0078173	PP2 dissociation from (STAT1N*)2
kf28	-0.537823	0.00530863	STAT1 deactivation by PP2 within (STAT1N*)2
kf29	8.67E-07	-3.84E-07	STAT1N combination with STAT1N*
kb29	-0.00022888	1.85E-05	STAT1N dissociation from STAT1N*

kf30	-0.474515	-6.65E-07	STAT1C binding at (IFN-R-JAK)2*-SOCS1 complex
kb30	0.543759	-8.42E-08	STAT1C dissociation from (IFN-R-JAK)2*-SOCS1 complex
kf31	-0.349744	-4.44E-07	SHP2 binding at (IFN-R-JAK)2*-STAT1C-SOCS1 complex
kb31	0.34494	-3.07E-07	SHP2 dissociation from (IFN-R-JAK)2*-STAT1C-SOCS1 complex
kf32	-0.229267	-8.59E-08	(IFN-R-JAK)2*-STAT1C-SHP2-SOCS1 complex dissociation
kf33	-0.0576678	-4.44E-07	SOCS1 dissociation from (IFN-R-JAK)2*-STAT1C-SHP2-SOCS1 complex
n_kf1	0.00590598	1.85E-07	STAT3 binding at IFNR sites
n_kr1	-0.00416891	-3.92E-07	STAT3 dissociation from IFNR sites
n_k2	0.00402307	-6.10E-07	STAT3 activation rate at IFNR sites
n_kf3	-6.42E-06	-3.55E-07	STAT3C* binding at IFNR sites
n_kr3	6.53E-06	-8.21E-08	STAT3C* dissociation from IFNR sites
n_kf4	2.76E-07	0.100181	STAT1 binding at gp130 sites
n_kr4	2.76E-07	-0.0591942	STAT1 dissociation from gp130 sites
n_k5	2.42E-07	0.0640334	STAT1 activation rate at gp130 sites
n_kf6	-2.42E-07	-9.89E-06	STAT1C* binding at gp130 sites
n_kr6	1.79E-11	7.80E-06	STAT1C* dissociation from gp130 sites
n_kf7	1.46E-07	-0.00103274	Max rate of (STAT1N*)2 inducing mRNA-SOCS3
n_kr7	2.59E-07	0.000793487	Km of (STAT1N*)2 inducing mRNA -SOCS3
n_kf8	0.00461257	0.00504897	STAT1/3 dimers formation in cytosol
n_kr8	-0.0042064	-0.00436441	STAT1/3 dimers dissociation in cytosol
n_kf9	-0.021741	-0.0671042	STAT1/3 dimers formation in nucleus
n_kr9	0.0264869	0.0613915	STAT1/3 dimers dissociation in nucleus
n_k10	0.00187114	-0.00882142	STAT1/3 dimers nuclear import
n_kf11	-0.0052467	-0.0085852	PP1 combination with STAT1/3 dimers
n_kr11	0.00408078	0.00669567	PP1 dissociation from STAT1/3 dimers
n_kf12	0.0244101	-0.103754	PP2 combination with STAT1/3 dimers
n_kr12	-0.0223063	0.101852	PP2 dissociation from STAT1/3 dimers

n_k13	-0.00882717	-0.00158213	STAT1 inactivation by PP1 within STAT1/3 dimers
n_k14	0.00130904	-0.012887	STAT3 inactivation by PP1 within STAT1/3 dimers
n_k15	-0.196276	0.082933	STAT1 inactivation by PP2 within STAT1/3 dimers
n_k16	0.0742326	-0.423821	STAT3 inactivation by PP2 within STAT1/3 dimers
n_kf17	0.175374	3.28E-09	STAT1 binding at IFNR sites
n_kr17	-0.12568	0	STAT1 dissociation from IFNR sites
n_kf18	0.117156	0	STAT3 binding at IFNR sites
n_kr18	-0.10681	0	STAT3 dissociation from IFNR sites
n_k19	0.316427	0	STAT1 binding at gp130 sites
n_k20	-0.05966	0	STAT1 dissociation from gp130 sites
n_kf21	1.70E-06	0.36268	STAT3 binding at gp130 sites
n_kr21	1.70E-06	-0.27986	STAT3 dissociation from gp130 sites
n_kf22	1.16E-06	0.010229	STAT1 activation rate at IFNR sites
n_kr22	0	-0.00879	STAT3 activation rate at IFNR sites
n_k23	3.38E-11	0.019239	STAT1 activation rate at gp130 sites
n_k24	3.22E-07	0.365773	STAT3 activation rate at gp130 sites

The definition of the parameter sensitivity can refer to the "Methods" section in the main text. The positive (or negative) sensitivity values indicate consistent (or opposite) percentage changes in output to the perturbation in the parameter compared to the nominal solution.

New biochemical reactions added to the crosstalk model

$$\left(IFN\text{-}R\text{-}JAK\right)2^* + STAT3C \underset{n\_kr1}{\overset{n\_kf1}{\rightleftarrows}} \left(IFN\text{-}R\text{-}JAK\right)2^*\text{-}STAT3C \quad \text{(N1)}$$

$$\left(IFN\text{-}R\text{-}JAK\right)2^*\text{-}STAT3C \xrightarrow{n\_k2} \left(IFN\text{-}R\text{-}JAK\right)2^* + STAT3C^* \quad \text{(N2)}$$

$$\left(IFN\text{-}R\text{-}JAK\right)2^* + STAT3C^* \underset{n\_kr3}{\overset{n\_kf3}{\rightleftarrows}} \left(IFN\text{-}R\text{-}JAK\right)2^*\text{-}STAT3C^* \quad \text{(N3)}$$

$$\left(IL6\text{-}gp80\text{-}gp130\text{-}JAK\right)2^* + STAT1C \underset{n\_kr4}{\overset{n\_kf4}{\rightleftarrows}} \left(IL6\text{-}gp80\text{-}gp130\text{-}JAK\right)2^*\text{-}STAT1C \quad \text{(N4)}$$

$$\left(IL6\text{-}gp80\text{-}gp130\text{-}JAK\right)2^*\text{-}STAT1C \xrightarrow{n\_k5} \left(IL6\text{-}gp80\text{-}gp130\text{-}JAK\right)2^* + STAT1C^* \quad \text{(N5)}$$

$$\left(IL6\text{-}gp80\text{-}gp130\text{-}JAK\right)2^* + STAT1C^* \underset{n\_kr6}{\overset{n\_kf6}{\rightleftarrows}} \left(IL6\text{-}gp80\text{-}gp130\text{-}JAK\right)2^*\text{-}STAT1C^* \quad \text{(N6)}$$

$$d\left[mRNA\text{-}SOCS3N\right]/dt = V\max\left(STAT1N*\right)2/\left[\left(STAT1N*\right)2 + Km\right] \quad \text{(N7)}$$

$$STAT1C* + STAT3C* \underset{n\_kr8}{\overset{n\_kf8}{\rightleftarrows}} STAT1C*\text{-}STAT3C* \quad \text{(N8)}$$

$$STAT1N* + STAT3N* \underset{n\_kr9}{\overset{n\_kf9}{\rightleftarrows}} STAT1N*\text{-}STAT3N* \quad \text{(N9)}$$

$$STAT1C*\text{-}STAT3C* \xrightarrow{n\_k10} STAT1N*\text{-}STAT3N* \quad \text{(N10)}$$

$$PP1 + STAT1C*\text{-}STAT3C* \underset{n\_kr11}{\overset{n\_kf11}{\rightleftarrows}} PP1\text{-}STAT1C*\text{-}STAT3C* \quad \text{(N11)}$$

$$PP2 + STAT1N*\text{-}STAT3N* \underset{n\_kr12}{\overset{n\_kf12}{\rightleftarrows}} PP2\text{-}STAT1N*\text{-}STAT3N* \quad \text{(N12)}$$

$$PP1\text{-}STAT1C*\text{-}STAT3C* \xrightarrow{n\_k13} PP1 + STAT1C + STAT3C* \quad \text{(N13)}$$

$$PP1\text{-}STAT1C*\text{-}STAT3C* \xrightarrow{n\_k14} PP1 + STAT1C* + STAT3C \quad \text{(N14)}$$

$$PP2\text{-}STAT1N*\text{-}STAT3N* \xrightarrow{n\_k15} PP2 + STAT1N + STAT3N* \quad \text{(N15)}$$

$$PP2\text{-}STAT1N*\text{-}STAT3N* \xrightarrow{n\_k16} PP2 + STAT1N* + STAT3N \quad \text{(N16)}$$

Where *R* represents receptor; *C* represents species within cytoplasm; *N* represents species within nuclei; 2 represents dimers; and * represents activation state. The *mRNA-SOCS3N* represents SOCS3 mRNA in the nucleus; *(STAT1N*)2* represents phosphorylated STAT1 homodimers in the nucleus; V_{max} represents maximum reaction velocity of the SOCS3-mRNA transcription; K_m represents half of substrate concentration when enzymatic reaction gets the maximum reaction velocity.

New biochemical reactions added to the non-competitive model

$$\left(IFN\text{-}R\text{-}JAK\right)2*\text{-}STAT3C + STAT1C \underset{n\_kr17}{\overset{n\_kf17}{\rightleftarrows}} \left(IFN\text{-}R\text{-}JAK\right)2*\text{-STAT1C-}STAT3C \quad \text{(N17)}$$

$$\left(IFN\text{-}R\text{-}JAK\right)2*\text{-}STAT1C + STAT3C \underset{n\_kr18}{\overset{n\_kf18}{\rightleftarrows}} \left(IFN\text{-}R\text{-}JAK\right)2*\text{-STAT1C-}STAT3C \quad \text{(N18)}$$

$$\left(IL6\text{-}gp80\text{-}gp130\text{-}JAK\right)2*\text{-}STAT3C + STAT1C \underset{n\_kr19}{\overset{n\_kf19}{\rightleftarrows}} \left(IL6\text{-}gp80\text{-}gp130\text{-}JAK\right)2*\text{-}STAT1C\text{-}STAT3C \quad \text{(N19)}$$

$$\left(IL6\text{-}gp80\text{-}gp130\text{-}JAK\right)2*\text{-}STAT1C + STAT3C \underset{n\_kr20}{\overset{n\_kf20}{\rightleftarrows}} \left(IL6\text{-}gp80\text{-}gp130\text{-}JAK\right)2*\text{-}STAT1C\text{-}STAT3C \quad \text{(N20)}$$

$$\left(IFN\text{-}R\text{-}JAK\right)2*\text{-}STAT1C\text{-}STAT3C \xrightarrow{n\_k21} \left(IFN\text{-}R\text{-}JAK\right)2*\text{-}STAT3C + STAT1C* \quad \text{(N21)}$$

$$\left(IFN\text{-}R\text{-}JAK\right)2*\text{-}STAT1C\text{-}STAT3C \xrightarrow{n\_k22} \left(IFN\text{-}R\text{-}JAK\right)2*\text{-}STAT1C + STAT3C* \quad \text{(N22)}$$

$$\left(IL6\text{-}gp80\text{-}gp130\text{-}JAK\right)2*\text{-}STAT1C\text{-}STAT3C \xrightarrow{n\_k23} \left(IFN\text{-}R\text{-}JAK\right)2*\text{-}STAT3C + STAT1C* \quad \text{(N23)}$$

$$\left(IL6\text{-}gp80\text{-}gp130\text{-}JAK\right)2*\text{-}STAT1C\text{-}STAT3C \xrightarrow{n\_k24} \left(IFN\text{-}R\text{-}JAK\right)2*\text{-}STAT1C + STAT3C* \quad \text{(N24)}$$

Biochemical reactions of IFNγ signalling part of the crosstalk model

$$R + JAK_{(IFN)} \underset{kb1}{\overset{kf1}{\rightleftarrows}} R\text{-}JAK \quad \text{(V1)}$$

$$IFN + R\text{-}JAK \underset{kb2}{\overset{kf2}{\rightleftarrows}} IFN\text{-}R\text{-}JAK \quad \text{(V2)}$$

$$(IFN\text{-}R\text{-}JAK) + (IFN\text{-}R\text{-}JAK) \underset{kb3}{\overset{kf3}{\rightleftarrows}} (IFN\text{-}R\text{-}JAK)2 \quad \text{(V3)}$$

$$(IFN\text{-}R\text{-}JAK)2 \xrightarrow{kf4} (IFN\text{-}R\text{-}JAK)2^* \quad \text{(V4)}$$

$$(IFN\text{-}R\text{-}JAK)2^* + STAT1C \underset{kb5}{\overset{kf5}{\rightleftarrows}} (IFN\text{-}R\text{-}JAK)2^*\text{-}STAT1C \quad \text{(V5)}$$

$$(IFN\text{-}R\text{-}JAK)2^*\text{-}STAT1C \xrightarrow{kf6} (IFN\text{-}R\text{-}JAK)2^* + STAT1C^* \quad \text{(V6)}$$

$$(IFN\text{-}R\text{-}JAK)2^* + STAT1C^* \underset{kb7}{\overset{kf7}{\rightleftarrows}} (IFN\text{-}R\text{-}JAK)2^*\text{-}STAT1C^* \quad \text{(V7)}$$

$$STAT1C^* + STAT1C^* \underset{kb8}{\overset{kf8}{\rightleftarrows}} (STAT1C^*)2 \quad \text{(V8)}$$

$$(IFN\text{-}R\text{-}JAK)2^* + SHP2 \underset{kb9}{\overset{kf9}{\rightleftarrows}} (IFN\text{-}R\text{-}JAK)2^*\text{-}SHP2 \quad \text{(V9)}$$

$$(IFN\text{-}R\text{-}JAK)2^*\text{-}SHP2 \xrightarrow{kf10} (IFN\text{-}R - JAK)2 + SHP2 \quad \text{(V10)}$$

$$PP1 + STAT1C^* \underset{kb11}{\overset{kf11}{\rightleftarrows}} PP1\text{-}STAT1C^* \quad \text{(V11)}$$

$$PP1\text{-}STAT1C^* \xrightarrow{kf12} PP1 + STAT1C \quad \text{(V12)}$$

$$STAT1C + STAT1C^* \underset{kb13}{\overset{kf13}{\rightleftarrows}} STAT1C\text{-}STAT1C^* \quad \text{(V13)}$$

$$(STAT1C^*)2 \overset{kf14}{\rightleftarrows} (STAT1N^*)2 \quad \text{(V14)}$$

$$PP2 + STAT1N^* \underset{kb15}{\overset{kf15}{\rightleftarrows}} PP2\text{-}STAT1N^* \quad \text{(V15)}$$

$$PP2\text{-}STAT1N^* \xrightarrow{kf16} PP2 + STAT1N \quad \text{(V16)}$$

$$STAT1N \xrightarrow{kf17} STAT1C \quad \text{(V17)}$$

$$d[mRNA\text{-}SOCS1N]/dt = k18a * (STAT1N^*)2/[(STAT1N^*)2 + k18b] \quad \text{(V18)}$$

$$mRNA\text{-}SOCS1N \xrightarrow{kf19} mRNA\text{-}SOCS1C \quad \text{(V19)}$$

$$d[SOCS1]/dt = kf20 * mRNA\text{-}SOCS1C \quad \text{(V20)}$$

$$(IFN\text{-}R\text{-}JAK)2^* + SOCS1 \underset{kb21}{\overset{kf21}{\rightleftarrows}} (IFN\text{-}R\text{-}JAK)2^*\text{-}SOCS1 \quad \text{(V21)}$$

$$d[mRNA\text{-}SOCS1C]/dt = -kf22 * mRNA\text{-}SOCS1C \quad \text{(V22)}$$

$$d[SOCS1C]/dt = -kf23 * SOCS1C \qquad \text{(V23)}$$

$$PP1 + (STAT1C^*)2 \underset{kb24}{\overset{kf24}{\rightleftarrows}} PP1\text{-}(STAT1C^*)2 \qquad \text{(V24)}$$

$$PP1\text{-}(STAT1C^*)2 \xrightarrow{kf25} PP1 + STAT1C\text{-}STAT1C^* \qquad \text{(V25)}$$

$$STAT1N^* + STAT1N^* \underset{kb26}{\overset{kf26}{\rightleftarrows}} (STAT1N^*)2 \qquad \text{(V26)}$$

$$PP2 + (STAT1N^*)2 \underset{kb27}{\overset{kf27}{\rightleftarrows}} PP2\text{-}(STAT1N^*)2 \qquad \text{(V27)}$$

$$PP2\text{-}(STAT1N^*)2 \xrightarrow{kf28} PP2 + STAT1N\text{-}STAT1N^* \qquad \text{(V28)}$$

$$STAT1N + STAT1N^* \underset{kb29}{\overset{kf29}{\rightleftarrows}} STAT1N\text{-}STAT1N^* \qquad \text{(V29)}$$

$$(IFN\text{-}R\text{-}JAK)2^*\text{-}SOCS1 + STAT1C \underset{kb30}{\overset{kf30}{\rightleftarrows}} (IFN\text{-}R\text{-}JAK)2^*\text{-}STAT1C\text{-}SOCS1 \qquad \text{(V30)}$$

$$(IFN\text{-}R\text{-}JAK)2^*\text{-}STAT1C\text{-}SOCS1 + SHP2 \underset{kb31}{\overset{kf31}{\rightleftarrows}} (IFN\text{-}R\text{-}JAK)2^*\text{-}STAT1C\text{-}SHP2\text{-}SOCS1 \qquad \text{(V31)}$$

$$(IFN\text{-}R\text{-}JAK)2^*\text{-}STAT1C\text{-}SHP2\text{-}SOCS1 \xrightarrow{kf32} (IFN\text{-}R\text{-}JAK)2^* + SOCS1 + STAT1C + SHP2 \qquad \text{(V32)}$$

$$(IFN\text{-}R\text{-}JAK)2^*\text{-}SOCS1\text{-}STAT1C\text{-}SHP2 \xrightarrow{kf33} (IFN\text{-}R\text{-}JAK)2^*\text{-}STAT1C\text{-}SHP2 + SOCS1 \qquad \text{(V33)}$$

Biochemical reactions of IL-6 signalling part of the crosstalk model

$$IL6 + gp80 \underset{k1r}{\overset{k1f}{\rightleftarrows}} IL6\text{-}gp80 \qquad (1)$$

$$gp130 + JAK_{(IL\text{-}6)} \underset{k2r}{\overset{k2f}{\rightleftarrows}} gp130\text{-}JAK \qquad (2)$$

$$IL6\text{-}gp80 + gp130\text{-}JAK \underset{k3r}{\overset{k3f}{\rightleftarrows}} IL6\text{-}gp80\text{-}gp130\text{-}JAK \qquad (3)$$

$$(IL6\text{-}gp80\text{-}gp130\text{-}JAK) + (IL6\text{-}gp80\text{-}gp130\text{-}JAK) \underset{k6r}{\overset{k6f}{\rightleftarrows}} (IL6\text{-}gp80\text{-}gp130\text{-}JAK)2 \qquad (6)$$

$$(IL6\text{-}gp80\text{-}gp130\text{-}JAK)2 \xrightarrow{k7} (IL6\text{-}gp80\text{-}gp130\text{-}JAK)2^* \qquad (7)$$

$$(IL6\text{-}gp80\text{-}gp130\text{-}JAK)2^* + STAT3C \underset{k8r}{\overset{k8f}{\rightleftarrows}} (IL6\text{-}gp80\text{-}gp130\text{-}JAK)2^*\text{-}STAT3C \qquad (8)$$

$$(IL6\text{-}gp80\text{-}gp130\text{-}JAK)2^*\text{-}STAT3C \xrightarrow[k9r]{k9f} (IL6\text{-}gp80\text{-}gp130\text{-}JAK)2^* + STAT3C^* \qquad (9)$$

$$(IL6\text{-}gp80\text{-}gp130\text{-}JAK)2^* + STAT3C^* \underset{k10r}{\overset{k10f}{\rightleftarrows}} (IL6\text{-}gp80\text{-}gp130\text{-}JAK)2^*\text{-}STAT3C^* \qquad (10)$$

$$STAT3C^* + STAT3C^* \underset{k16r}{\overset{k16f}{\rightleftarrows}} (STAT3C^*)2 \qquad (16)$$

$$(IL6\text{-}gp80\text{-}gp130\text{-}JAK)2^* + SHP2 \underset{k17r}{\overset{k17f}{\rightleftarrows}} (IL6\text{-}gp80\text{-}gp130\text{-}JAK)2^*\text{-}SHP2 \qquad (17)$$

$$(IL6\text{-}gp80\text{-}gp130\text{-}JAK)2^*\text{-}SHP2 \xrightarrow{k18} (IL6\text{-}gp80\text{-}gp130\text{-}JAK)2 + SHP2 \quad (18)$$

$$PP1 + STAT3C^* \underset{k19r}{\overset{k19f}{\rightleftarrows}} PP1\text{-}STAT3C^* \quad (19)$$

$$PP1\text{-}STAT3C^* \xrightarrow{k20} PP1 + STAT3C \quad (20)$$

$$PP1 + (STAT3C^*)2 \underset{k21r}{\overset{k21f}{\rightleftarrows}} PP1\text{-}(STAT3C^*)2 \quad (21)$$

$$PP1\text{-}(STAT3C^*)2 \underset{k22r}{\overset{k22f}{\rightleftarrows}} PP1 + STAT3C\text{-}STAT3C^* \quad (22)$$

$$STAT3C + STAT3C^* \underset{k23r}{\overset{k23f}{\rightleftarrows}} STAT3C\text{-}STAT3C^* \quad (23)$$

$$(STAT3C^*)2 \xrightarrow{k24} (STAT3N^*)2 \quad (24)$$

$$(STAT3N^*)2 \underset{k25r}{\overset{k25f}{\rightleftarrows}} STAT3N^* + STAT3N^* \quad (25)$$

$$PP2 + STAT3N^* \underset{k26r}{\overset{k26f}{\rightleftarrows}} PP1\text{-}STAT3N^* \quad (26)$$

$$PP2\text{-}STAT3N^* \xrightarrow{k27} PP2 + STAT3N \quad (27)$$

$$PP2 + (STAT3N^*)2 \underset{k28r}{\overset{k28f}{\rightleftarrows}} PP2\text{-}(STAT3N^*)2 \quad (28)$$

$$PP2\text{-}(STAT3N^*)2 \underset{k29r}{\overset{k29f}{\longrightarrow}} PP2 + STAT3N\text{-}STAT3N^* \quad (29)$$

$$STAT3N\text{-}STAT3N^* \underset{k30r}{\overset{k30f}{\rightleftarrows}} STAT3N + STAT3N^* \quad (30)$$

$$STAT3N \xrightarrow{k31} STAT3C \quad (31)$$

$$d[mRNA\text{-}SOCS3N]/dt = k32f * (STAT3N^*)2/[(STAT3N^*)2 + k32r] \quad (32)$$

$$mRNA-SOCS3N \xrightarrow{k33} mRNA-SOCS3C \quad (33)$$

$$d[SOCS3]/dt = k34 * mRNA\text{-}SOCS3C \quad (34)$$

$$(IL6\text{-}gp80\text{-}gp130\text{-}JAK)2^* + SOCS3 \underset{k35r}{\overset{k35f}{\rightleftarrows}} (IL6\text{-}gp80\text{-}gp130\text{-}JAK)2^*\text{-}SOCS3 \quad (35)$$

$$d[mRNA\text{-}SOCS3C]/dt = -k36 * mRNA\text{-}SOCS3C \quad (36)$$

$$d[SOCS3C]/dt = -k37 * SOCS3C \quad (37)$$

$$(IL6\text{-}gp80\text{-}gp130\text{-}JAK)2^*\text{-}SOCS3 \xrightarrow{k38} (IL6\text{-}gp80\text{-}gp130\text{-}JAK)2 + SOCS3 \quad (38)$$

$$(IL6\text{-}gp80\text{-}gp130\text{-}JAK)2^*\text{-}SHP2 \underset{k39r}{\overset{k39f}{\rightleftarrows}} (IL6\text{-}gp80\text{-}gp130\text{-}JAK)2^*\text{-}SHP2^* \quad (39)$$

$$\left(IL6\text{-}gp80\text{-}gp130\text{-}JAK\right)2^*\text{-}SHP2^* + Grb2 \underset{k40r}{\overset{k40f}{\rightleftarrows}} \left(IL6\text{-}gp80\text{-}gp130\text{-}JAK\right)2^*\text{-}SHP2^*\text{-}Grb2 \quad (40)$$

$$\left(IL6\text{-}gp80\text{-}gp130\text{-}JAK\right)2^*\text{-}SHP2^*\text{-}Grb2 + SOS \underset{k41r}{\overset{k41f}{\rightleftarrows}} \left(IL6\text{-}gp80\text{-}gp130\text{-}JAK\right)2^*\text{-}SHP2^*\text{-}Grb2\text{-}SOS \quad (41)$$

$$\left(IL6\text{-}gp80\text{-}gp130\text{-}JAK\right)2^*\text{-}SHP2^*\text{-}Grb2\text{-}SOS + Ras\text{-}GDP \underset{k42r}{\overset{k42f}{\rightleftarrows}} \left(IL6\text{-}gp80\text{-}gp130\text{-}JAK\right)2^*\text{-}SHP2^*\text{-}Grb2\text{-}SOS\text{-}Ras\text{-}GDP \quad (42)$$

$$\left(IL6\text{-}gp80\text{-}gp130\text{-}JAK\right)2^*\text{-}SHP2^*\text{-}Grb2\text{-}SOS\text{-}Ras\text{-}GDP \underset{k43r}{\overset{k43f}{\rightleftarrows}} \left(IL6\text{-}gp80\text{-}gp130\text{-}JAK\right)2^*\text{-}SHP2^*\text{-}Grb2\text{-}SOS + Ras\text{-}GTP \quad (43)$$

$$Ras\text{-}GTP + Raf \xrightarrow{k44} Raf\text{-}Ras\text{-}GTP \quad (44)$$

$$Raf\text{-}Ras\text{-}GTP \xrightarrow{k45} Ras\text{-}GTP^* + Raf^* \quad (45)$$

$$\left(IL6\text{-}gp80\text{-}gp130\text{-}JAK\right)2^*\text{-}SHP2^*\text{-}Grb2\text{-}SOS + Ras\text{-}GTP^* \underset{k46r}{\overset{k46f}{\rightleftarrows}} \left(IL6\text{-}gp80\text{-}gp130\text{-}JAK\right)2^*\text{-}SHP2^*\text{-}Grb2\text{-}SOS\text{-}Ras\text{-}GTP \quad (46)$$

$$\left(IL6\text{-}gp80\text{-}gp130\text{-}JAK\right)2^*\text{-}SHP2^*\text{-}Grb2\text{-}SOS\text{-}Ras\text{-}GTP \underset{k47r}{\overset{k47f}{\rightleftarrows}} \left(IL6\text{-}gp80\text{-}gp130\text{-}JAK\right)2^*\text{-}SHP2^*\text{-}Grb2\text{-}SOS + Ras\text{-}GDP \quad (47)$$

$$\left(IL6\text{-}gp80\text{-}gp130\text{-}JAK\right)2^*\text{-}SHP2^*\text{-}Grb2\text{-}SOS \underset{k48r}{\overset{k48f}{\rightleftarrows}} \left(IL6\text{-}gp80\text{-}gp130\text{-}JAK\right)2^* + SHP2^*\text{-}Grb2\text{-}SOS \quad (48)$$

$$SHP2^*\text{-}Grb2\text{-}SOS \underset{k49r}{\overset{k49f}{\rightleftarrows}} Grb2\text{-}SOS + SHP2^* \quad (49)$$

$$Grb2\text{-}SOS \underset{k50r}{\overset{k50f}{\rightleftarrows}} Grb2 + SOS \quad (50)$$

$$SHP2^* \xrightarrow{k51} SHP2 \quad (51)$$

$$\left(IL6\text{-}gp80\text{-}gp130\text{-}JAK\right)2^*\text{-}SHP2^* \underset{k52r}{\overset{k52f}{\rightleftarrows}} \left(IL6\text{-}gp80\text{-}gp130\text{-}JAK\right)2^* + SHP2^* \quad (52)$$

$$Grb2 + SHP2^* \underset{k53r}{\overset{k53f}{\rightleftarrows}} SHP2^*\text{-}Grb2 \quad (53)$$

$$\left(IL6\text{-}gp80\text{-}gp130\text{-}JAK^*\right)2\text{-}SHP2^*\text{-}Grb2 \underset{k54r}{\overset{k54f}{\rightleftarrows}} \left(IL6\text{-}gp80\text{-}gp130\text{-}JAK^*\right)2 + SHP2^*\text{-}Grb2 \quad (54)$$

$$SOS + SHP2^*\text{-}Grb2 \underset{k55r}{\overset{k55f}{\rightleftarrows}} SHP2^*\text{-}Grb2\text{-}SOS \quad (55)$$

$$\left(IL6\text{-}gp80\text{-}gp130\text{-}JAK\right)2^*\text{-}SHP2^* + Grb2\text{-}SOS \underset{k56r}{\overset{k56f}{\rightleftarrows}} \left(IL6\text{-}gp80\text{-}gp130\text{-}JAK\right)2^*\text{-}SHP2^*\text{-}Grb2\text{-}SOS \quad (56)$$

$$Phosp1 + Raf^* \underset{k57r}{\overset{k57f}{\rightleftarrows}} Raf^*\text{-}Phosp1 \quad (57)$$

$$Raf^*\text{-}Phosp1 \xrightarrow{k58} Raf + Phosp1 \quad (58)$$

$$Raf^* + MEK \underset{k59r}{\overset{k59f}{\rightleftarrows}} MEK\text{-}Raf^* \quad (59)$$

$$MEK\text{-}Raf^* \xrightarrow{k60} MEK\text{-}P + Raf^* \quad (60)$$

$$MEK\text{-}P + Raf^* \underset{k61r}{\overset{k61f}{\rightleftarrows}} MEK\text{-}P\text{-}Raf^* \quad (61)$$

$$MEK\text{-}P\text{-}Raf^* \xrightarrow{k62} MEK\text{-}PP + Raf^* \quad (62)$$

$$MEK\text{-}PP + Phosp2 \underset{k63r}{\overset{k63f}{\rightleftarrows}} MEK\text{-}PP\text{-}Phosp2 \quad (63)$$

$$MEK\text{-}PP\text{-}Phosp2 \xrightarrow{k64} MEK\text{-}P + Phosp2 \quad (64)$$

$$MEK\text{-}P + Phosp2 \underset{k65r}{\overset{k65f}{\rightleftarrows}} MEK\text{-}P\text{-}Phosp2 \quad (65)$$

$$MEK\text{-}P\text{-}Phosp2 \xrightarrow{k66} MEK + Phosp2 \quad (66)$$

$$MEK\text{-}PP + ERK \underset{k67r}{\overset{k67f}{\rightleftarrows}} ERK\text{-}MEK\text{-}PP \quad (67)$$

$$ERK - MEK - PP \xrightarrow{k68} ERK - P + MEK - PP \quad (68)$$

$$ERK\text{-}P + MEK\text{-}PP \underset{k69r}{\overset{k69f}{\rightleftarrows}} ERK\text{-}P\text{-}MEK\text{-}PP \quad (69)$$

$$ERK\text{-}P\text{-}MEK\text{-}PP \xrightarrow{k70} ERK\text{-}PP + MEK\text{-}PP \quad (70)$$

$$ERK\text{-}PP + Phosp3 \underset{k71r}{\overset{k71f}{\rightleftarrows}} ERK\text{-}PP\text{-}Phosp3 \quad (71)$$

$$ERK\text{-}PP\text{-}Phosp3 \xrightarrow{k72} ERK\text{-}P + Phosp3 \quad (72)$$

$$ERK\text{-}P + Phosp3 \underset{k73r}{\overset{k73f}{\rightleftarrows}} ERK\text{-}P\text{-}Phosp3 \quad (73)$$

$$ERK\text{-}P\text{-}Phosp3 \xrightarrow{k74} ERK + Phosp3 \quad (74)$$

$$d[CEBP]/dt = k75f * CEBPi * ERK\text{-}PP/(CEBPi + k75r) \quad (75)$$

$$CEBP \xrightarrow{k76} CEBPi \quad (76)$$

$$CEBP + CEBP \underset{k77r}{\overset{k77f}{\rightleftarrows}} CEBPn \quad (77)$$

Reference

1. Yamada S, Shiono S, Joo A, Yoshimura A: **Control mechanism of JAK/STAT signal transduction pathway**. *FEBS Lett* 2003, **534**(1-3):190-196.
2. Moya C, Huang Z, Cheng P, Jayaraman A, Hahn J: **Investigation of IL-6 and IL-10 signalling via mathematical modelling**. *IET Syst Biol* 2011, **5**(1):15.
